# Three *Toxoplasma gondii* Dense Granule Proteins Are Required for Induction of Lewis Rat Macrophage Pyroptosis

**DOI:** 10.1128/mBio.02388-18

**Published:** 2019-01-08

**Authors:** Yifan Wang, Kimberly M. Cirelli, Patricio D. C. Barros, Lamba Omar Sangaré, Vincent Butty, Musa A. Hassan, Patricia Pesavento, Asli Mete, Jeroen P. J. Saeij

**Affiliations:** a Department of Pathology, Microbiology and Immunology, School of Veterinary Medicine, University of California, Davis, Davis, California, USA; b Department of Biology, Massachusetts Institute of Technology, Cambridge, Massachusetts, USA; c College of Medicine and Veterinary Medicine, The University of Edinburgh, Edinburgh, United Kingdom; d The Roslin Institute, The University of Edinburgh, Edinburgh, United Kingdom; e Center for Tropical Livestock Health and Genetics, The University of Edinburgh, Edinburgh, United Kingdom; UC Irvine

**Keywords:** Dense granule proteins, macrophages, NLRP1 inflammasomes, pyroptosis, *Toxoplasma gondii*

## Abstract

Upon invasion of Lewis rat macrophages, *Toxoplasma* rapidly induces programmed cell death (pyroptosis), which prevents *Toxoplasma* replication, possibly explaining the resistance of the Lewis rat to *Toxoplasma*. Using a chemical mutagenesis screen, we identified *Toxoplasma* mutants that no longer induced pyroptosis. Whole-genome sequencing led to the identification of three *Toxoplasma* parasitophorous vacuole-localized dense granule proteins, GRA35, GRA42, and GRA43, that are individually required for induction of Lewis rat macrophage pyroptosis. Macrophage infection with Δ*gra35*, Δ*gra42*, and Δ*gra43* parasites led to greatly reduced cell death rates and enhanced parasite replication. Lewis rat macrophages infected with parasites containing a single, double, or triple deletion of these GRAs showed similar levels of cell viability, suggesting that the three GRAs function in the same pathway. Deletion of *GRA42* or *GRA43* resulted in GRA35 (and other GRAs) being retained inside the parasitophorous vacuole instead of being localized to the parasitophorous vacuole membrane. Despite having greatly enhanced replication in Lewis rat macrophages *in vitro*, Δ*gra35*, Δ*gra42*, and Δ*gra43* parasites did not establish a chronic infection in Lewis rats. *Toxoplasma* did not induce F344 rat macrophage pyroptosis, but F344 rats infected with Δ*gra35*, Δ*gra42*, and Δ*gra43* parasites had reduced cyst numbers. Thus, these GRAs determined parasite *in vivo* fitness in F344 rats. Overall, our data suggest that these three *Toxoplasma* dense granule proteins play a critical role in establishing a chronic infection *in vivo*, independently of their role in mediating macrophage pyroptosis, likely due to their importance in regulating protein localization to the parasitophorous vacuole membrane.

## Abstract (precis)

Inflammasomes are major components of the innate immune system and are responsible for detecting various microbial and environmental danger signals. Upon invasion of Lewis rat macrophages, the parasite rapidly activates the NLRP1 inflammasome, resulting in pyroptosis and elimination of the parasite’s replication niche. The work reported here revealed that *Toxoplasma* GRA35, GRA42, and GRA43 are required for induction of Lewis rat macrophage pyroptosis. GRA42 and GRA43 mediate the correct localization of other GRAs, including GRA35, to the parasitophorous vacuole membrane. These three GRAs were also found to be important for parasite *in vivo* fitness in a *Toxoplasma*-susceptible rat strain, independently of their role in NLRP1 inflammasome activation, suggesting that they perform other important functions. Thus, this study identified three GRAs that mediate the induction of Lewis rat macrophage pyroptosis and are required for pathogenesis of the parasite.

## INTRODUCTION

*Toxoplasma* is an obligate intracellular protozoan parasite that infects a wide variety of warm-blooded animals (1). Among its different hosts, there are natural differences in susceptibility to the parasite. Most laboratory mouse strains are susceptible to infection and can succumb after low-dose injection of virulent parasite strains. Rats and humans are relatively resistant to *Toxoplasma*. Most rat strains remain asymptomatic after infection, but the parasite establishes a chronic infection by developing into cysts in brain and muscle tissues. However, the Lewis rats can clear the parasite, leading to failure to develop a chronic infection (2). This resistance was shown to be a myeloid cell-intrinsic dominant trait that mapped to the *Toxo1* locus containing *Nlrp1* (nucleotide-binding oligomerization domain, leucine-rich repeat protein 1), which encodes for the NLRP1 inflammasome sensor (3, 4). *In vitro*, Lewis rat bone marrow-derived macrophages (BMDMs) are sensitive to *Toxoplasma*-induced cell death and secrete mature interleukin-1β (IL-1β) (4, 5). Because Lewis rat macrophages die rapidly upon *Toxoplasma* invasion, parasites are released into the extracellular space before replication can occur (4, 5). We and others previously established, using different inbred rat strains and recombinant inbred lines derived from crosses between resistant and susceptible rats, that there is a perfect correlation between sensitivity to *Toxoplasma*-induced macrophage cell death and decreased parasite proliferation, IL-1β/IL-18 processing, rat resistance to *Toxoplasma* infection, and NLRP1 sequence (4–6). Furthermore, we have shown that macrophage cell death is mediated by Lewis rat NLRP1 as knockdown of *Nlrp1* inhibited cell death whereas expression of the Lewis *Nlrp1* allele in F344 rat macrophages made them susceptible to *Toxoplasma*-induced cell death (5).

The inflammasomes are a family of cytosolic pattern recognition receptors (PRRs). Activation of the sensor leads to the formation of a multimeric complex and the recruitment and proteolytic activation of pro-caspase-1. Caspase-1 cleaves the cytokines pro-IL-1β and pro-IL-18, resulting in their release from cells. Active caspase-1 also cleaves gasdermin D (GSDMD), which can subsequently form pores in the host cell membrane and is therefore an essential trigger for a type of host cell death termed pyroptosis (7, 8). Pyroptosis is a highly inflammatory form of programmed cell death that occurs most frequently upon infection with intracellular pathogens and has been established as a host mechanism to promote the rapid clearance of various microbial infections by removing their intracellular replication niche (9). As macrophages are among the predominant cell types that are infected upon *Toxoplasma* infection (10), it is possible that macrophage pyroptosis is a host mechanism to prevent parasite proliferation inside the host. Furthermore, the cytokines released from pyroptotic macrophages might attract other immune cells to fight the infection. Infected macrophages and dendritic cells are also involved in promoting *Toxoplasma* dissemination by migrating to distant sites (11–13), and therefore *Toxoplasma*-induced pyroptosis of these cells could also inhibit *Toxoplasma* dissemination.

The specific stimuli that can activate the NLRP1 inflammasome resulting in pyroptosis and their mechanisms of activation differ. Anthrax lethal toxin (LT) is a protease and a direct activator of rat NLRP1 (14). LT cleaves the N terminus of NLRP1 in LT-susceptible mouse and rat macrophages. This cleavage is sufficient to activate the NLRP1 inflammasome and induce pyroptosis (15, 16). Val-boroPro (VbP), a nonselective inhibitor of post-proline cleaving serine proteases, activates the NLRP1 inflammasome and triggers pyroptosis of monocytes and macrophages via inhibition of the cytosolic serine dipeptidases Dpp8 and Dpp9 (17, 18). However, unlike what was shown for LT, VbP activation of the NLRP1 inflammasome does not involve direct proteolysis (18). NLRP1 inflammasome activation by *Toxoplasma* in mice was also recently evaluated (6, 19). No cleavage of the mouse NLRP1 was observed in parasite-infected cells, suggesting that the NLRP1 response to *Toxoplasma* in mice might be independent of cleavage (6). However, the parasite protein(s) involved in activation of the NLRP1 inflammasome is unknown.

To further explore the mechanism of activation of the Lewis rat NLRP1 inflammasome by *Toxoplasma*, we chose to take an unbiased approach to identify the *Toxoplasma* gene product(s) required for induction of Lewis rat BMDM pyroptosis. Using a chemical mutagenesis screen followed by whole-genome sequencing, we identified three *Toxoplasma* dense granule proteins (GRA35, GRA42, and GRA43) that are required for induction of Lewis rat macrophage pyroptosis. Parasite strains deficient in GRA35, GRA42, or GRA43 induce significantly less pyroptosis and IL-1β processing and secretion but have enhanced replication. These results indicate that *Toxoplasma* dense granule proteins are involved in NLRP1 inflammasome activation.

## RESULTS

### The NLRP3 inflammasome is not involved in *Toxoplasma*-induced Lewis rat macrophage pyroptosis.

**FIG 1:**
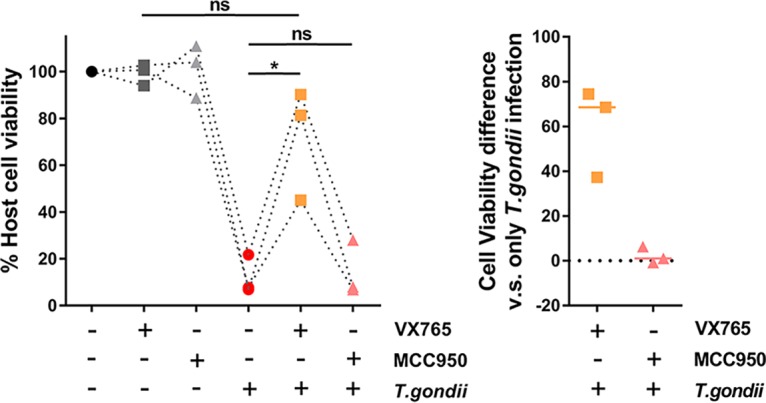
The NLRP3 inflammasome is dispensable for *Toxoplasma*-induced Lewis rat macrophage cell death and IL-1β secretion. Lewis rat BMDMs with or without pretreatment with either 50 μM VX765 or 10 μM MCC950 for 2 h were subjected to infection with *Toxoplasma* type I (RH) parasites (MOI = 0.5) for 24 h. Macrophage viability was measured via 3-(4,5-dimethylthiazol-2-yl)-5-(3-carboxymethoxyphenyl)-2-(4-sulfophenyl)-2H-tetrazolium (MTS) assay. Data are displayed as paired scatterplots (left; *n* = 3; *, *P < *0.05; ns, not significant [Student’s *t* test]). The right scatterplots show the cell viability difference between infected BMDMs with and without treatment in each paired experiment. Horizontal bars represent the median cell viability difference.

We previously showed that *Toxoplasma* activates the NLRP1 inflammasome in Lewis rat macrophages, resulting in pyroptosis (5). *Toxoplasma* activates both the NLRP1 and NLRP3 inflammasomes in mice (19), but it is not known whether *Toxoplasma* also activates the NLRP3 inflammasome in Lewis rat macrophages. To investigate this, Lewis rat macrophages were treated with the NLRP3 inflammasome inhibitor MCC950 (20) or with the caspase-1 inhibitor VX765 (which should inhibit all inflammasomes) (21) followed by *Toxoplasma* type I (RH) parasite infection. Infected macrophages treated with VX765 showed significantly higher cell viability than nontreated macrophages, whereas treatment with MCC950 did not prevent parasite-induced cell death (Fig. 1). VX765 and MCC950 did not inhibit parasite invasion in Lewis rat macrophages (Fig. S1A) or parasite growth in human foreskin fibroblasts (HFFs) (Fig. S1B). As a positive control, MCC950 inhibited cell death and IL-1β release in response to nigericin, a known NLRP3 agonist, in lipopolysaccharide (LPS)-primed Lewis rat macrophages (Fig. S1C and D). Therefore, Lewis rat macrophage cell death upon *Toxoplasma* infection is likely entirely dependent on NLRP1.

10.1128/mBio.02388-18.1 FIG S1 Neither caspase-1 inhibitor nor NLRP3 inflammasome inhibitor affects parasite invasion. (A) Lewis rat BMDMs (1 × 10^5^) were incubated with or without 50 μM of VX765 or 10 μM of MCC950 for 2 h followed by infection with 1 × 10^5^ of *Toxoplasma* type I (RH) parasites for 30 minutes. Data represent quantification of levels of the invading and invaded parasites per host nucleus. Data are displayed as average values with scatterplots for each independent experiment (*n *=* *3; error bars, ± SD; ns, not significant [one-way ANOVA with Kruskal-Wallis test]). (B) Confluent HFFs were incubated with or without VX765 (50 μM) or MCC950 (10 μM) followed by infection with parasites for 4 days. The area of at least 40 plaques per experiment was measured. Data are displayed as average values with scatterplots for each independent experiment (*n* = 2; error bars, ± SD; ns, not significant [one-way ANOVA with Kruskal-Wallis test]). (C) Lewis rat BMDMs primed with 100 ng/ml LPS for 2 h were treated with 10 μM of MCC950 for 2 h or left untreated followed by incubation with or without 10 μM nigericin for 2 h. Macrophage viability was measured via MTS assay. Data are displayed as paired scatterplots (*n* =* *3; *, *P < *0.05; **, *P < *0.01; ns, not significant [Student’s *t* test]). (D) IL-1β secretion was measured using ELISA on the cell supernatants from the experiments described in the panel C legend. Data are displayed as paired scatterplots (*n* = 3; *, *P < *0.05; ns, not significant [Student’s *t* test]). Download FIG S1, TIF file, 0.3 MB. Copyright © 2019 Wang et al. 2019 Wang et al. This content is distributed under the terms of the Creative Commons Attribution 4.0 International license.

### Pyroptosis of Lewis rat macrophages is dependent on *Toxoplasma* Golgi-protease ASP5 but not on the MYR1 translocon.

**FIG 2:**
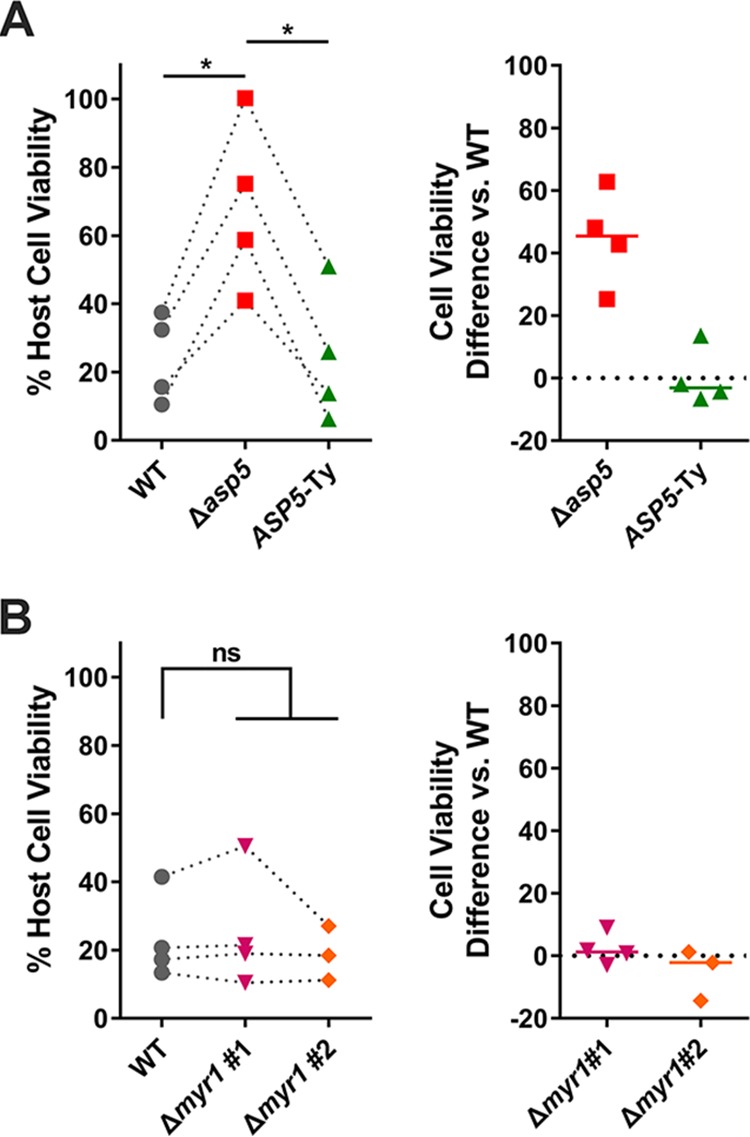
*Toxoplasma*-induced Lewis rat macrophage cell death is ASP5 dependent but not MYR1 dependent. (A) Lewis rat BMDMs were infected with WT parasites, ASP5 knockout parasites (Δ*asp5*), or ASP5 knockout parasites complemented with a Ty-tagged copy of *ASP5* (*ASP5*-Ty) (MOI = 1) for 24 h. Macrophage viability was measured via MTS assay. Data are displayed as paired scatterplots (left; *n* = 4; *, *P < *0.05 [Student’s *t* test]). The right scatterplots show the cell viability difference between the indicated strains and WT parasites in each paired experiment. Horizontal bars represent the median cell viability difference. (B) Lewis rat BMDMs were infected with WT parasites or two independent clones of MYR1 knockout parasites (Δ*myr1* #1 and Δ*myr1* #2) (MOI = 1) for 24 h. Macrophage viability was measured via MTS assay. Data are displayed as paired scatterplots (left; *n* = 4 for WT and Δ*myr1* #1 and *n* = 3 for Δ*myr1* #2; ns, not significant [Student's *t* test]). The right scatterplots show the cell viability difference between Δ*myr1* parasites and WT parasites in each paired experiment. Horizontal bars represent the median cell viability difference.

To better understand the mechanism of NLRP1 inflammasome activation, we aimed to discover the *Toxoplasma* protein(s) that induces Lewis rat macrophage pyroptosis. We focused on parasite secretory proteins that can potentially interact with host cytosolic NLRP1 or with other host cytosolic proteins that modulate the activity of the inflammasome. Upon invasion, *Toxoplasma* secretes rhoptry proteins (ROPs) into the host cell cytosol (22). We previously showed that parasites treated with mycalolide B, a compound that blocks *Toxoplasma* invasion but allows secretion of microneme and rhoptry contents, were unable to induce Lewis rat macrophage IL-1β secretion and cell death (5), suggesting that ROPs are not the parasite effectors that activate the NLRP1 inflammasome. Once the parasite resides inside a host cell in a nonfusogenic parasitophorous vacuole (PV), dense granules discharge GRAs into the PV lumen, where some stay while others eventually become associated with the PV membrane (PVM) or are exported into the host cytosol (23). *Toxoplasma* aspartyl protease 5 (ASP5), a Golgi-resident protease related to *Plasmodium* plasmepsin V, mediates the export of GRAs to the host cytosol and can influence the localization of several GRAs to the PVM (24–26). To investigate whether GRAs that localize at the PVM or GRAs that are exported to the host cytosol induce Lewis macrophage cell death, the viability of Lewis rat macrophages infected with Δ*asp5* parasites was measured (Fig. 2A). Infection by Δ*asp5* parasites induced less macrophage cell death than wild-type (WT) parasite infection, and Δ*asp5* parasites complemented with a Ty-tagged copy of *ASP5* regained the ability to induce cell death (Fig. 2A, right panel). MYR1, a putative *Toxoplasma* PVM translocon, mediates the export of GRAs, including GRA16 and GRA24, into the host cytosol (27). Δ*myr1* parasites (Fig. S2A and C) induced levels of Lewis rat macrophage cell death similar to those seen with WT parasites (Fig. 2B). Taking the data together, *Toxoplasma*-induced Lewis rat macrophage cell death is ASP5 but not MYR1 dependent, suggesting that GRAs that localize to the PVM, but not GRAs exported to the host cytosol, are likely mediators of Lewis macrophage pyroptosis.

10.1128/mBio.02388-18.2 FIG S2 PCR confirming knockout of candidate genes. (A) Schematic diagram depicting the genomic loci of the genes of interest (GOI) (top) and the CRISPR/Cas9-targeting site (red box). Linearized pTKOatt plasmid containing HXGPRT selection cassettes (middle) was used as a repair template to disrupt GOI loci (bottom) after mycophenolic acid and xanthine selection. P1 and P2 refer to primers used for checking locus disruption. (B) Schematic diagram depicting the strategy used for making the double/triple knockouts. The *GRA42* locus or the *GRA43* locus or both loci in Δ*gra35* parasites were disrupted by CRISPR/Cas9 cleavage, and a linearized pLoxp-DHFR-mCherry plasmid containing a DHFR-TS selection cassette was used for nonhomologous end joining (NHEJ) repair of the double-stranded break. After pyrimethamine selection and limiting dilution, single clones with DHFR-mCherry integrated into the *GRA42* locus and an intact *GRA43* locus were used as the Δ*gra35* Δ*gra42* strain; single clones with an intact *GRA42* locus and DHFR-mCherry integrated into the *GRA43* locus were used as the Δ*gra35* Δ*gra43* strain; single clones with DHFR-mCherry integrated into both loci were used as the triple knockout strain. (C) Genomic DNA was isolated from clones and used as the template. Knockout was determined by failure to amplify the gene of interest using P1 and P2 as primers. DNA quality was assessed by amplifying *TGGT1_309160* (i, ii, iii, and vi [for Δ*myr1* and single, double, and triple knockout of *GRA35*, *GRA42*, and *GRA43*, respectively]), the *B1* gene (ii [for Δ*TGGT1_248260*]), or the GRA35 gene (iii and iv [for Δ*TGGT1_203040* and Δ*rop17*, respectively]). Download FIG S2, TIF file, 0.6 MB. Copyright © 2019 Wang et al. 2019 Wang et al. This content is distributed under the terms of the Creative Commons Attribution 4.0 International license.

### Isolation of *Toxoplasma* mutants that do not induce Lewis macrophage pyroptosis.

**FIG 3:**
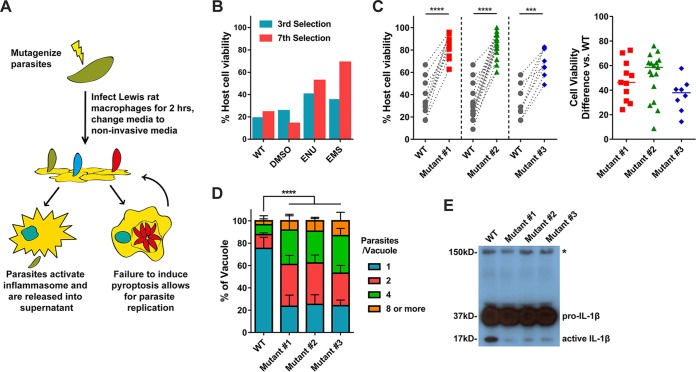
Isolation of *Toxoplasma* mutants that do not induce Lewis rat macrophage cell death. (A) Schematic of mutagenesis screen. DS, dextran sulfate; BMDMs, bone marrow-derived macrophages. (B) Lewis rat BMDMs were infected with indicated mutagenized parasites (MOI = 1) for 24 h. Macrophage viability was measured via MTS assay. Data are displayed in columns (*n* = 1). (C) Lewis rat BMDMs were infected with WT parasites or independent mutant strains isolated from the pool of mutagenized parasites (Mutant #1, Mutant #2, and Mutant #3) (MOI = 1) for 24 h. Macrophage viability was measured via MTS assay. Data are displayed as paired scatterplots (left; *n *≥* *8 for WT, *n* = 11 for mutant 1, *n* = 17 for mutant 2, *n* = 8 for mutant 3; ***, *P < *0.001; ****, *P < *0.0001 [Student's *t* test]). The right scatterplots show the cell viability difference between the indicated mutant strains and WT parasites in each paired experiment. Horizontal bars represent the median cell viability difference. (D) Lewis rat BMDMs were infected with the strains used as described for panel C (MOI = 0.5) for 24 h. The number of parasites per vacuole was quantified by microscopy. A total of 100 to 120 vacuoles were counted per experiment. Data are displayed as average values (*n* = 4; error bars, + standard deviations [SD]; ****, *P < *0.0001 [two-way analysis of variance {ANOVA} comparing mutants to WT]). (E) Western blot probing for IL-1β on concentrated (20×) supernatants of LPS-primed (100 ng/ml, 2 h) Lewis rat BMDMs infected with the strains used as described for panel C (MOI = 1) for 24 h. The image is representative of results from two experiments; pro-IL-1β is 37 kDa; active IL-1β is 17 kDa; the aspecific band is represented by an asterisk and indicates similar levels of loading of samples.

Although GRAs that localize to the PVM are likely involved in Lewis macrophage pyroptosis, the exact protein(s) involved is still unknown. To identify the *Toxoplasma* gene product(s) required for activation of Lewis macrophage pyroptosis, we designed a chemical mutagenesis screen to isolate mutants that fail to induce cell death (Fig. 3A). Type I (RH) parasites were mutagenized by the use of *N-*ethyl-*N-*nitrosourea (ENU) or ethyl methanesulfonate (EMS). The populations of chemically mutagenized parasites were used to infect Lewis rat macrophages at a multiplicity of infection (MOI) of 0.2 to 0.3. *Toxoplasma*-induced pyroptosis is a dominant trait (5); reinvasion of parasites into the rare cells containing *Toxoplasma* mutants that do not activate pyroptosis would therefore still lead to macrophage cell death. Therefore, to inhibit reinvasion, extracellular parasites were washed from cells after 2 h of infection and the medium was replaced with fresh medium that contained the glycosaminoglycan dextran sulfate (DS), a glycan competitor that prevents host cell invasion by extracellular parasites (28). Parasites that retained the ability to induce cell death were released from the lysed cell into the supernatant, where the parasite was coated with DS, blocking reinvasion into a new host cell. Mutated parasites unable to induce cell death were able to replicate within the surviving macrophage. After 24 h of infection, the surviving cells were washed, thereby removing the extracellular parasites capable of inducing cell death from the population. The surviving macrophages were then added to a monolayer of human foreskin fibroblasts (HFFs) so the parasites within the macrophages could continue to replicate until their natural egress from the macrophages.

After seven rounds of selection, a distinct phenotype (the level of viability of Lewis rat macrophages upon *Toxoplasma* infection is more than 50%) began to emerge in two independent populations of mutagenized parasites compared to WT and dimethyl sulfoxide (DMSO)-treated parasites (Fig. 3B). After a further two rounds of selection, single parasites were cloned from the populations and individual clones were tested for their inability to induce pyroptosis. Three independent mutant clones induced significantly less Lewis rat macrophage cell death (Fig. 3C). Macrophage survival was linked to the ability of the parasite to replicate within the macrophage. As expected, 75% of the surviving macrophages infected with WT parasites contained only single parasites whereas only 25% of cells infected with the mutants contained single parasites (Fig. 3D). Cell death mediated by inflammasome activation (pyroptosis) is characterized by active IL-1β secretion. We found a strong decrease in the amount of cleaved active IL-1β (17 kDa) secreted from macrophages infected with each of the mutant strains compared to WT results (Fig. 3E). Thus, the forward genetic selection strategy was successful in yielding *Toxoplasma* mutants deficient in the induction of Lewis rat macrophage pyroptosis.

### Identification of single nucleotide variations in the mutants.

**FIG 4:**
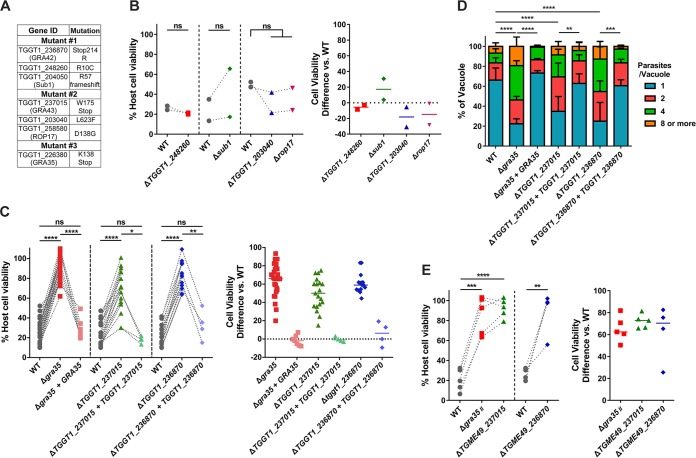
Three genes are individually required to induce cell death in Lewis rat BMDMs. (A) List of genes containing nonsynonymous polymorphisms that fulfill candidate gene criteria in isolated mutants. (B) Lewis rat BMDMs were infected with WT parasites or with parasites in which *TGGT1_248260*, *SUB1*, *TGGT1_203040*, or *ROP17* was knocked out (Δ*TGGT1_248260*, Δ*sub1*, Δ*TGGT1_203040*, or Δ*rop17*) (MOI = 1) for 24 h. Macrophage viability was measured via MTS assay. Data are displayed on the left as paired scatterplots (*n* = 2; ns, not significant [Student's *t* test]). The right scatterplots show the cell viability difference between the indicated knockout strains and WT parasites in each paired experiment. Horizontal bars represent the median cell viability difference. (C) Cell viability as assessed by MTS assay of Lewis rat BMDMs infected with WT parasites or with parasites in which *GRA35*, *TGGT1_237015*, or *TGGT1_236870* was knocked out (Δ*gra35*, Δ*TGGT1_237015*, or Δ*TGGT1_236870*) or with knockout parasites complemented with WT alleles of *GRA35*, *TGGT1_237015*, or *TGGT1_236870* (Δ*gra35 *+* GRA35*, Δ*TGGT1_237015 + TGGT1_237015*, or Δ*TGGT1_236870 + TGGT1_236870*) (MOI = 1) for 24 h. Data are displayed on the left as paired scatterplots (*n *≥* *16 for WT, *n* = 28 for Δ*gra35*, *n* = 7 for Δ*gra35 *+* GRA35*, *n* = 19 for Δ*TGGT1_237015*, *n* = 4 for Δ*TGGT1_237015 *+* TGGT1_237015*, *n* = 16 for Δ*TGGT1_236870*, *n* = 4 for Δ*TGGT1_236870 *+* TGGT1_236870*; *, *P < *0.05; **, *P < *0.01; ****, *P < *0.0001; ns, not significant [Student's *t* test]). The right scatterplots show the cell viability difference between the indicated strains and WT parasites in each paired experiment. Horizontal bars represent the median cell viability difference. (D) Number of parasites per vacuole were measured in Lewis rat BMDMs infected with the strains used as described for panel C (MOI = 0.5) at 24 h postinfection. A total of 100 to 120 vacuoles were counted per experiment. Data are displayed as average values (*n* = 5 for WT and Δ*TGGT1_237015*, *n* = 4 for Δ*gra35*, *n* = 3 for Δ*TGGT1_236870*, *n* = 2 for all the complementation strains; error bars, + SD; **, *P < *0.01; ***, *P < *0.001; ****, *P < *0.0001 [two-way ANOVA multiple comparisons]). (E) Lewis rat BMDMs were infected with type II WT parasites or type II parasites in which *GRA35*, *TGME49_237015*, or *TGME49_236870* was knocked out (Δ*gra35_II_*, Δ*TGME49_237015*, or Δ*TGME49_236870*) (MOI = 1) for 24 h. Macrophage viability was measured via MTS assay. Data are displayed as paired scatterplots (left; *n *≥* *4 for WT; *n* = 5 for Δ*gra35_II_* and Δ*TGME49_237015*; *n* = 4 for Δ*TGME49_236870*; **, *P < *0.01; ***, *P < *0.001; ****, *P < *0.0001 [Student's *t* test]). The right scatterplots show the cell viability difference between the indicated knockout strains and WT parasites in each paired experiment. Horizontal bars represent the median cell viability difference.

To identify the genes mutated in each clone, we performed whole-genome sequencing of each mutant. Sequence comparisons relative to the parental strain revealed 16, 11, and 12 nonsynonymous mutations in mutant 1, mutant 2, and mutant 3, respectively (Table 1). The three mutants did not have any mutated genes in common. To identify the causative mutations in these mutants, we established a set of criteria to shorten the list of possible genes. The inflammasomes are expressed and assembled within the cytoplasm of host cells. We therefore chose to focus on those *Toxoplasma* genes whose protein products contained predicted signal peptides. Additionally, we had previously tested a large number of different *Toxoplasma* strains for their ability to activate cell death (5); all strains tested were able to induce cell death. We therefore focused on the genes that were expressed (fragments per kilobase per million [FPKM] of >10) across all strains based on our published transcriptome sequencing (RNA-seq) data set for these strains (29). Using these criteria, we shortened the list of candidate genes in these mutants to seven (Fig. 4A).

**TABLE 1:** List of all identified nonsynonymous mutations^*a*^

Chromosome	Position	Ref	Sub	Codon change	AA change	Gene	Mut no.
TGGT1_chrXII	3698939	C	T	Cgt/Tgt	R/C	TGGT1_248260	1
TGGT1_chrXI	4323464	A	G	cTc/cCc	L/P	TGGT1_314875	1
TGGT1_chrX	5454719	A	T	Tga/Aga	*/R	TGGT1_236870	1
TGGT1_chrVIII	3546892	A	G	Aca/Gca	T/A	TGGT1_273510	1
TGGT1_chrVIIb	258249	C	G	Ccg/Gcg	P/A	TGGT1_263360	1
TGGT1_chrVIIb	1300287	A	G	tTc/tCc	F/S	TGGT1_262825	1
TGGT1_chrVIIb	4053654	G	C	Ccg/Gcg	P/A	TGGT1_257500	1
TGGT1_chrVIIa	683027	A	G	Ttc/Ctc	F/L	TGGT1_206550	1
TGGT1_chrVIIa	1666878	A	G	tTg/tCg	L/S	TGGT1_204310	1
TGGT1_chrV	2683109	A	C	Ttg/Gtg	L/V	TGGT1_284040	1
TGGT1_chrIX	1745808	G	A	cCc/cTc	P/L	TGGT1_264890	1
TGGT1_chrIX	3803976	T	C	Tct/Cct	S/P	TGGT1_290960	1
TGGT1_chrIII	527809	A	T	aaA/aaT	K/N	TGGT1_252395	1
TGGT1_chrIII	1241431	C	T	Gac/Aac	D/N	TGGT1_253870	1
TGGT1_chrIb	814454	A	T	cTg/cAg	L/Q	TGGT1_208580	1
TGGT1_chrVIIa	2153702	GA	A	GAg/Aga	E/R	TGGT1_204050	1
TGGT1_chrIV	2235861	G	A	cGa/cAa	R/Q	TGGT1_301250	2
TGGT1_chrVIIa	2964132	C	G	ttG/ttC	L/F	TGGT1_203040	2
TGGT1_chrVI	290424	T	C	Aaa/Gaa	K/E	TGGT1_239130	2
TGGT1_chrVI	3356628	G	C	Gga/Cga	G/R	TGGT1_243635	2
TGGT1_chrV	121175	A	G	aTc/aCc	I/T	TGGT1_220175	2
TGGT1_chrXII	5959927	A	C	cAt/cCt	H/P	TGGT1_278518	2
TGGT1_chrVIII	2061823	T	C	Agt/Ggt	S/G	TGGT1_231410	2
TGGT1_chrVIIb	730342	C	T	Cgt/Tgt	R/C	TGGT1_264140	2
TGGT1_chrVIIb	2573674	G	A	cCa/cTa	P/L	TGGT1_260450	2
TGGT1_chrVIIb	3451345	A	G	gAt/gGt	D/G	TGGT1_258580	2
TGGT1_chrX	5567109	C	T	tGg/tAg	W/*	TGGT1_237015	2
TGGT1_chrIX	2023395	T	C	gAa/gGa	E/G	TGGT1_264472	3
TGGT1_chrV	1043175	T	C	Acg/Gcg	T/A	TGGT1_213610	3
TGGT1_chrVI	1514625	A	T	gAt/gTt	D/V	TGGT1_240960	3
TGGT1_chrVI	674303	C	T	aGa/aAa	R/K	TGGT1_239700	3
TGGT1_chrVIIa	1197377	C	G	Gcc/Ccc	A/P	TGGT1_205160	3
TGGT1_chrVIII	2566377	G	T	gaG/gaT	E/D	TGGT1_233120	3
TGGT1_chrX	1583637	A	T	Aaa/Taa	K/*	TGGT1_226380	3
TGGT1_chrX	3027043	T	A	Agc/Tgc	S/C	TGGT1_224280	3
TGGT1_chrX	401396	T	C	Agt/Ggt	S/G	TGGT1_228210	3
TGGT1_chrXI	2517037	A	G	cAc/cGc	H/R	TGGT1_312140	3
TGGT1_chrXII	1102624	T	C	Aag/Gag	K/E	TGGT1_219070	3
TGGT1_chrXII	6691803	T	C	aAg/aGg	K/R	TGGT1_277030	3

a AA, amino acid; Ref, reference nucleotide(s) in WT strain (GT1 v9.0); Sub, nucleotide variant(s); Mut, mutant clone number; *, stop codon.

To determine which of these genes are involved in activation of Lewis rat macrophage cell death, we individually disrupted each candidate gene in the RH background (Fig. S2A and C) and tested the resulting strains for their inability to induce cell death. Parasites in which we knocked out *TGGT1_248260*, *SUB1*, *TGGT1_203040*, or *ROP17* induced levels of cell death similar to those induced by WT parasites (Fig. 4B). Mutant 3 has only one candidate gene, *TGGT1_226380*, encoding GRA35 (30). A mutation in this gene resulted in an early stop codon (Fig. 4A; see also Fig. S3A). In mutant 2, a mutation in *TGGT1_237015* also resulted in an early stop codon (Fig. 4A; see also Fig. S3A). In mutant 1, a mutation in the stop codon of *TGGT1_236870* converted this stop codon into an arginine (R), which resulted in an extended gene product (Fig. 4A; see also Fig. S3A). Lewis rat macrophages infected with parasites that contained individual disruptions in *GRA35*, *TGGT1_237015*, or *TGGT1_236870* showed significantly less cell death than macrophages infected with WT parasites (Fig. 4C). Complementation of knockout strains with WT alleles of *GRA35*, *TGGT1_237015*, and *TGGT1_236870* restored their ability to induce cell death (Fig. 4C). The replication of Δ*gra35*, Δ*TGGT1_237015*, and Δ*TGGT1_236870* parasites in infected Lewis rat macrophages was significantly enhanced compared to the levels seen with WT parasites and complemented parasites 24 h after infection (Fig. 4D). Similarly, type II (ME49) parasites in which *GRA35*, *TGME49_237015*, or *TGME49_236870* was disrupted (Fig. S2C) induced lower levels of Lewis macrophage cell death than were seen with macrophages infected with WT parasites (Fig. 4E). We also sequenced these 3 genes in other independent mutants. Another mutation (Y121 mutant to stop codon) in *GRA35* was also found in one of these mutant clones (named mutant 4), which failed to induce pyroptosis (Fig. S3A and S4). These results indicated that the gene products of *GRA35*, *TGGT1_237015*, and *TGGT1_236870* mediated *Toxoplasma*-induced Lewis rat macrophage cell death.

10.1128/mBio.02388-18.3 FIG S3 Predicted structure and synonymous/nonsynonymous analysis of *GRA35*, *TGGT1_236870*, and *TGGT1_237015*. (A) The PSIPRED method was used for secondary structure prediction (Jones DT, J Mol Biol 292:195–202, 1999). Red stars indicate the mutation site in the amino acid sequence of mutants 1, 2, and 3. Blue stars indicate the *GRA35* mutation site in the amino acid sequence of mutant 4. Gray box, signal peptide; green box, α-helices. TmHMM2.0 was used for transmembrane domain (TM) prediction (Krogh A, et al., J Mol Biol 305:567–580, 2001). Blue box, TM. The coiled-coil domain was analyzed using http://www.ch.embnet.org/software/COILS_form.html (Lupas A, et al., Science 252:1162–1164, 1991). Yellow box, coiled-coil region. (B, C, and D) SNAP was used for synonymous/nonsynonymous analysis (https://www.hiv.lanl.gov/content/sequence/SNAP/SNAP.html) (Korber B, ch 4, p 55–72, *in* Rodrigo AH and Learn GH, ed, *Computational Analysis of HIV Molecular Sequences*, 2000). The coding sequences of *GRA35* (B), *TGGT1_236870* (C), and *TGGT1_237015* (D) from 64 strains (ToxoDB 29 release) were analyzed using SNAP V2.1.1. Download FIG S3, TIF file, 0.4 MB. Copyright © 2019 Wang et al. 2019 Wang et al. This content is distributed under the terms of the Creative Commons Attribution 4.0 International license.

10.1128/mBio.02388-18.4 FIG S4 Mutant 4 does not induce cell death in Lewis rat macrophages. Lewis rat BMDMs were infected with WT parasites or with mutant clone 4, which was isolated from the pool of mutagenized parasites (MOI = 1) for 24 h. Macrophage viability was measured via MTS assay. Data are displayed as paired scatterplots (left; *n *=* *4; **, *P < *0.01 [Student’s *t* test]). The right scatterplots show the cell viability difference between mutant 4 and the WT parasites in each paired experiment. Horizontal bars represent the median cell viability difference. Download FIG S4, TIF file, 0.5 MB. Copyright © 2019 Wang et al. 2019 Wang et al. This content is distributed under the terms of the Creative Commons Attribution 4.0 International license.

### *TGGT1_236870* and *TGGT1_237015* Code for novel PV-localized dense granule proteins.

GRA35 was identified as a novel PV-localized dense granule protein by Bio-ID using other GRAs as baits (30), but there are no reports on the gene products encoded by *TGGT1_237015* and *TGGT1_236870*. *GRA35*, *TGGT1_237015*, and *TGGT1_236870* are small one-exon genes that are expressed in all *Toxoplasma* life stages except in the sexual stages inside the cat (www.toxodb.org). The predicted protein products of these genes lack predicted functional domains except for the C-terminal coiled-coil domain of GRA35 (Fig. S3A). Each of the resulting proteins has a signal peptide and one predicted transmembrane (TM) domain and is generally predicted to be very alpha helical except the gene product of *TGGT1_236870* (Fig. S3A). No *Toxoplasma* export element (TEXEL [RRLxx]) motif (24) is present in the amino acid sequence of GRA35, TGGT1_237015, and TGGT1_236870. Although the corresponding three genes are quite highly conserved among different *Toxoplasma* strains, the rates of nonsynonymous/synonymous (NS/S) polymorphisms among 64 different strains are higher at the C terminus (starting after the TM domain) of each gene product (Fig. S3B to D). BLAST analysis of the entire protein sequence revealed no predicted function of these three genes. Orthologs of *GRA35*, *TGGT1_237015*, and *TGGT1_236870* were identified in other tissue cyst-forming coccidia, namely, Hammondia hammondi, Neospora caninum, and Besnoitia besnoiti (Fig. S5). We also found that three *Toxoplasma* proteins, TGGT1_225160, GRA36 (TGGT1_213067), and TGGT1_257970, shared high (>40%) amino acid similarity with GRA35 (Fig. S5A). Parasites deficient in *TGGT1_225160*, *GRA36*, or *TGGT1_257970* induced levels of Lewis rat macrophage cell death similar to those seen with infection with WT parasites, suggesting that these proteins do not share the GRA35 function that mediates Lewis rat macrophage cell death (Fig. S6).

10.1128/mBio.02388-18.5 FIG S5 GRA35, TGGT1_236870, and TGGT1_237015 have orthologues in *Hammondia*, *Neospora*, and *Besnoitia.* Alignments of primary peptide sequences were performed using PRALINE. (A) Alignment of GRA35 from type I, II, and III Toxoplasma gondii; Hammondia hammondi HHA_226380; Neospora caninum NCLIV_046580 and NCLIV_047520; Besnoitia besnoiti BESB_060230 and BESB_061290; and Toxoplasma gondii TGGT1_225160, GRA36, and TGGT1_257970. (B) Alignment of *Toxoplasma* TGGT1_236870, TGME49_236870, and TGVEG_236870; *Hammondia* HHA_236870; *Neospora* NCLIV_050780; and Besnoitia besnoiti BESB_036500. (C) Alignment of *Toxoplasma* TGGT1_237015, TGME49_237015, and TGVEG_237015; *Hammondia* HHA_237015; Neospora caninum NCLIV_050915; and Besnoitia besnoiti BESB_036360. Download FIG S5, TIF file, 2.9 MB. Copyright © 2019 Wang et al. 2019 Wang et al. This content is distributed under the terms of the Creative Commons Attribution 4.0 International license.

10.1128/mBio.02388-18.6 FIG S6 *GRA35* gene family members are not involved in *Toxoplasma*-induced cell death in Lewis rat BMDMs. (A) Schematic diagram depicting the genomic loci of GOI (top) and the CRISPR/Cas9-targeting site. Linearized pLoxp-DHFR-mCherry plasmid containing DHFR-TS selection cassettes (middle) was used as a repair template to disrupt GOI loci (bottom) after pyrimethamine selection. P1 and P2 refer to primers used for checking locus disruption; P1 and P3 refer to primers used for checking repair template integration. (B) PCR confirming individual knockout of *GRA35* gene family members with indicated primers. Genomic DNA isolated from each knockout parasites was used as the template. (C) Lewis rat BMDMs were infected with WT parasites or the parasites in which *GRA35*, *TGGT1_225160*, *GRA36*, or *TGGT1_257970* was knocked out (Δ*gra35*, Δ*TGGT1_225160*, Δ*gra36*, or Δ*TGGT1_257970*) (MOI = 1) for 24 h. Macrophage viability was measured via MTS assay. Data are displayed as paired scatterplots (left; *n *=* *3; all knockout strains versus WT) (**, *P < *0.01; ns, not significant [Student’s *t* test]). The right scatterplots show the cell viability difference between the indicated strains and WT parasites in each paired experiment. Horizontal bars represent the median cell viability difference. Download FIG S6, TIF file, 0.4 MB. Copyright © 2019 Wang et al. 2019 Wang et al. This content is distributed under the terms of the Creative Commons Attribution 4.0 International license.

**FIG 5:**
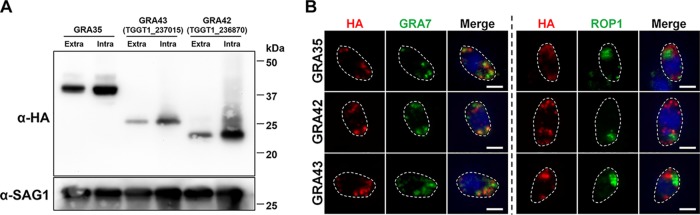
*TGGT1_236870* and *TGGT1_237015* code for novel dense granule proteins GRA42 and GRA43. (A) Strains individually knocked out in each gene were generated using CRISPR/Cas9 and complemented with an HA-tagged WT version of the gene. HFFs were infected with HA-expressing parasites for 24 h. Extracellular parasites were removed and washed with PBS prior to lysing (“Extra”). The remaining infected cells were lysed (“Intra”). SAG-1 was used as the parasite loading control. Predicted sizes: GRA35, 40.3 kDa; GRA42, 29.3 kDa; GRA43, 23.8 kDa. The image is representative of results from two independent experiments. (B) Extracellular parasites expressing HA-tagged GRA35, GRA42, or GRA43 were fixed, permeabilized, and subjected to immunofluorescent assay with the indicated antibodies. The images were taken at identical exposure times for each channel (scale bar = 2 μm). The image is representative of results from two independent experiments.

To characterize GRA35, TGGT1_237015, and TGGT1_236870, we generated complemented strains in which a C-terminally hemagglutinin (HA)-tagged version of each gene product is expressed from its endogenous promoter in the respective knockout strains. The expression of each protein was confirmed by Western blotting (Fig. 5A). The extracellular and intracellular parasites yielded bands migrating at the same size, suggesting that GRA35, TGGT1_237015, and TGGT1_236870 did not undergo proteolytic modification in the process of secretion. The subcellular localization of each protein was observed in extracellular parasites. As previously reported, GRA35 is a dense granule protein that localized at punctuate structures which overlapped GRA7 while being excluded from rhoptries (Fig. 5B). The gene products of *TGGT1_237015* and *TGGT1_236870* also showed colocalization with GRA7 but not with ROP1 (Fig. 5B). The three proteins were localized at the PVM and PV lumen in intracellular parasites (see Fig. 7B, upper row), suggesting that they are indeed secreted via dense granules. We concluded from these data that TGGT1_236870 and TGGT1_237015 are novel dense granule proteins; therefore, we named them GRA42 and GRA43, respectively.

### Complementation of mutants with GRA35, GRA42, and GRA43 restores their ability to induce Lewis rat macrophage pyroptosis.

**FIG 6:**
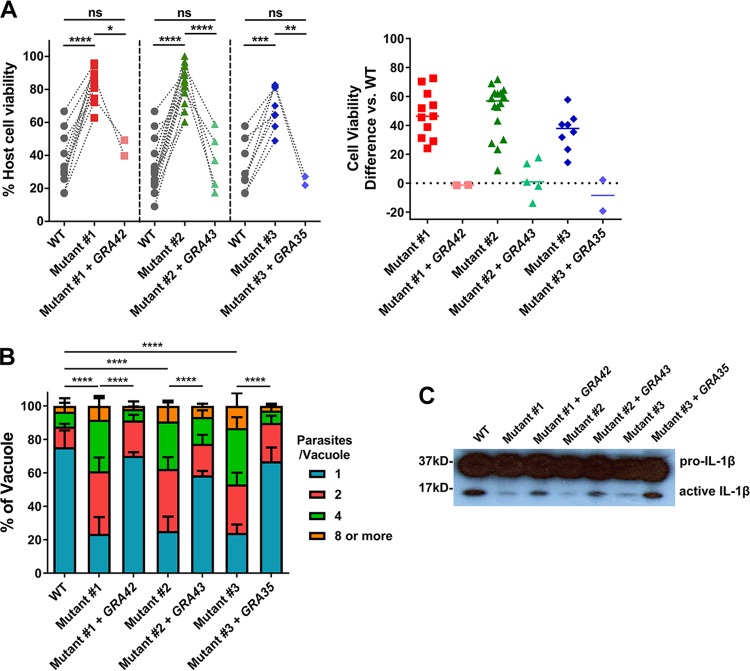
GRA35, GRA42, and GRA43 restore the mutant phenotype, and are required for inflammasome activation. (A) Lewis rat BMDMs were infected with WT parasites, with independent mutant strains isolated from the pool of mutagenized parasites (Mutant #1, Mutant #2 and Mutant #3), or with the mutant strains complemented with WT alleles of *GRA42*, *GRA43*, or *GRA35* (Mutant #1 + *GRA42*, Mutant #2 + *GRA43*, Mutant #3 + *GRA35*) (MOI = 1) for 24 h. Macrophage viability was measured via MTS assay. Data are displayed on the left as paired scatterplots (*n *≥* *8 for WT, *n* = 11 for mutant 1, *n* = 17 for mutant 2, *n* = 8 for mutant 3, *n* = 2 for mutant 1 + *GRA42* and mutant 3 + *GRA35*, *n* = 4 for mutant 2 + *GRA43*; *, *P < *0.05; **, *P < *0.01; ***, *P < *0.001; ****, *P < *0.0001 [Student's *t* test]). The right scatterplots show the cell viability difference between the indicated strains and WT parasites in each paired experiment. Horizontal bars represent the median cell viability difference. (B) The number of parasites per vacuole was measured in Lewis rat BMDMs infected with the strains used as described for panel A (MOI = 0.5) at 24 h postinfection. A total of 100 to 120 vacuoles were counted per experiment. Data are displayed as average values (*n* = 4 for the WT strain and mutants 1, 2, and 3; *n* = 2 for mutant 1 + *GRA42*, mutant 2 + *GRA43*, and mutant 3 + *GRA35*; error bars, + SD; ****, *P < *0.0001 [two-way ANOVA multiple comparisons]). (C) Western blot of IL-1β on concentrated supernatants (20×) BMDMs primed with LPS (100 ng/ml, 2 h) infected with the strains used as described for panel A (MOI =1, 24 h). The image is representative of results from two independent experiments.

To confirm that the mutation in GRA35, GRA42, and GRA43 was indeed responsible for the failure of our chemically mutagenized parasites to induce cell death, we expressed the WT allele of the gene in each mutant. Addition of the WT version of *GRA35*, *GRA42*, and *GRA43* to the respective mutants was sufficient to restore induction of cell death (Fig. 6A). Similarly, macrophages infected with mutant strains expressing the WT version of *GRA35*, *GRA42*, or *GRA43* contained fewer replicating parasites than the mutant-infected BMDMs (Fig. 6B). We also observed an increase in the level of active IL-1β secreted from macrophages infected with the complemented strains compared to their mutant counterparts (Fig. 6C). Overall, these data indicate that GRA35, GRA42, and GRA43 are required for induction of Lewis rat macrophage pyroptosis by *Toxoplasma*.

### GRA42 and GRA43 influence the correct localization of GRA35, and of other GRAs, to the PVM.

**FIG 7:**
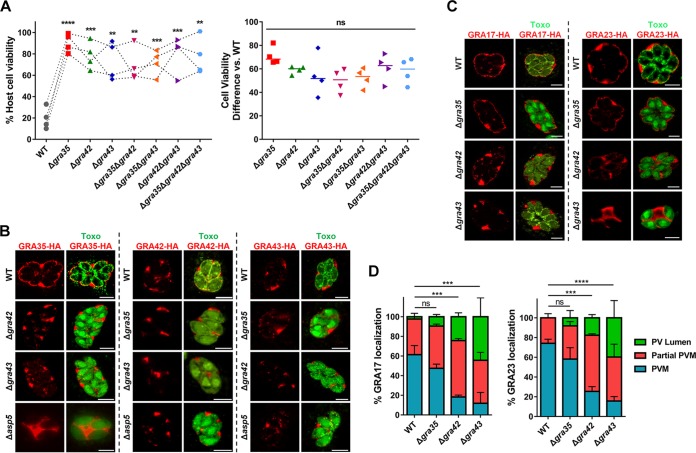
GRA42 and GRA43 influence the localization of GRA35, as well as GRA17, to the PVM. (A) Lewis rat BMDMs were infected with WT parasites or with parasites in which *GRA35*, *GRA42*, or *GRA43* was knocked out (Δ*gra35*, Δ*gra42*, or Δ*gra43*) or with parasites containing a double knockout of *GRA35*, *GRA42*, or *GRA43* (Δ*gra35* Δ*gra42*, Δ*gra35* Δ*gra43*, or Δ*gra42* Δ*gra43*) or with triple knockout parasites (Δ*gra35* Δ*gra42* Δ*gra43*) (MOI = 1) for 24 h. Macrophage viability was measured via MTS assay. Data are displayed as paired scatterplots (left; *n* = 4; all knockout strains versus WT; **, *P < *0.01; ***, *P < *0.001; ****, *P < *0.0001 [Student's *t* test]). The right scatterplots show the cell viability difference between the indicated strains and WT parasites in each paired experiment. Horizontal bars represent the median cell viability difference (ns, not significant [one-way ANOVA with Kruskal-Wallis test]). (B) HFFs were infected with WT parasites or with parasites in which *GRA35*, *GRA42*, *GRA43*, or *ASP5* was knocked out (Δ*gra35*, Δ*gra42*, Δ*gra43*, or Δ*asp5*) and that transiently expressed GRA35-HA (left), GRA42-HA (middle), or GRA43-HA (right). The parasites were fixed and stained with antibodies against the HA epitope (red) and SAG1 (green). Transfected parasites were GFP positive. Images were taken at identical exposure times for each channel (scale bar = 5 μm). The image is representative of results from two independent experiments. (C) HFFs were infected with WT parasites or with parasites in which *GRA42* or *GRA43* was knocked out (Δ*gra42* or Δ*gra43*) and that transiently expressed GRA17-HA (left) or GRA23-HA (right), fixed, and stained with antibodies against SAG1 (green) and the HA epitope (red). Transfected parasites were GFP positive. The images were taken at identical exposure times for each channel (scale bar = 5 μm). The image is representative of results from two independent experiments. (D) Localization of GRA17 or GRA23 (see panel C) in at least 60 vacuoles containing 4 or more parasites were observed and scored as PVM localization, partial PVM localization, or PV lumen localization. Data are displayed as average values (*n* = 2; error bars, + SD; ***, *P < *0.001; ****, *P < *0.0001 [two-way ANOVA comparing mutants to WT]).

Lewis rat macrophages infected with individual knockouts of *GRA35*, *GRA42*, or *GRA43* showed a level of reduced cell death similar to that shown by macrophages infected with WT parasites (Fig. 4C, right panel). It is therefore likely that these three GRAs function in the same pathway that induces cell death. To confirm this, we generated double and triple *GRA35*, *GRA42*, and *GRA43* knockout parasites (Fig. S2B and C). Single, double, and triple *GRA35*, *GRA42*, and *GRA43* knockout parasites induced similar levels of macrophage cell death (Fig. 7A), indicating that these GRAs function in the same pathway. Possibly, they form a protein complex that activates the inflammasome or one of the GRAs activates the inflammasome and the other two are upstream in the pathway. To investigate this possibility, we first determined the localization of GRA35, GRA42, and GRA43 in intracellular parasites expressing a C-terminally hemagglutinin (HA)-tagged version of each gene product driven by its endogenous promoter. GRA35 localized at the PVM, while GRA42 and GRA43 were predominantly localized in the PV lumen (Fig. 7B, upper row). We then determined the localization of GRA35, GRA42, and GRA43 in the different knockout parasites. In Δ*gra42* and Δ*gra43* parasites, GRA35 was mostly retained in the PV lumen and less of it was localized to the PVM, whereas the localization of GRA42 and GRA43 was unchanged regardless of the presence of GRA35, GRA42, or GRA43 (Fig. 7B, middle two rows). Previously, we had found that parasites deficient in ASP5 induced lower levels of Lewis rat macrophage cell death (Fig. 2A). *ASP5* deletion also resulted in mislocalization of certain PVM-localized GRAs (24, 25). To determine whether ASP5 might influence Lewis rat macrophage cell death through these GRAs, the localization of GRA35, GRA42, and GRA43 was also observed in parasites lacking ASP5. In Δ*asp5* parasites, GRA35 no longer localized to the PVM and was mostly present in the PV space (Fig. 7B, left bottom). In contrast, ASP5 did not influence the localization of GRA42 and GRA43 (Fig. 7B, middle and right bottom). Therefore, these results revealed that GRA42, GRA43, and ASP5 influenced the PVM localization of GRA35. To determine whether GRA35 is the only GRA whose localization is influenced by GRA42 and GRA43, we determined the localization of GRA17 and GRA23, which are also PVM-localized GRAs, in Δ*gra42* or Δ*gra43* parasites (Fig. 7C). In the WT parasites, these two GRAs were entirely localized at the PVM (Fig. 7C, top row). In the Δ*gra42* parasites, GRA17 and GRA23 were mislocalized to the PV space, although a small fraction localized to the PVM (Fig. 7C, middle row). In the Δ*gra43* parasites, these two GRAs were mostly absent at the PVM instead being retained in the PV lumen (Fig. 7C, bottom row). In contrast to GRA42 and GRA43, deficiency in GRA35 did not result in mislocalization of these two PVM GRAs. Note that only a small amount of GRA17 is required to mediate normal small-molecule permeability and to prevent enlarged vacuoles (31), possibly explaining why we had failed to see the established Δ*gra17* “bubble vacuole” phenotype in these vacuoles. Partial or no GRA17/GRA23 PVM staining was observed in more than 80% of the vacuoles of Δ*gra42* and Δ*gra43* parasites (Fig. 7D). Therefore, GRA42 and GRA43 not only influence GRA35 localization at the PVM but also affect the localization of other PVM-associated GRAs.

### No interaction between *Toxoplasma* GRA35 and Lewis rat NLRP1 in cotransfected HEK293T cells.

**FIG 8:**
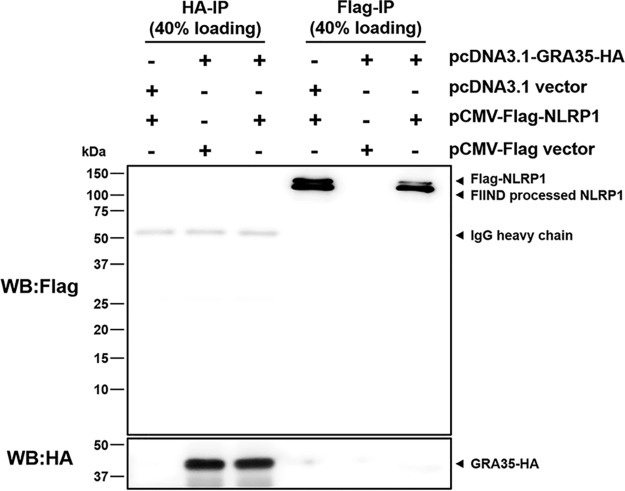
Lewis rat NLRP1 does not interact with *Toxoplasma* GRA35 in cotransfected HEK293T cells. HEK293T cells were cotransfected with pcDNA3.1-GRA35-HA and pCMV-FLAG-NLRP1 (expressing Lewis rat variant of Nlrp1) at a ratio of 1:1. At 30 h after transfection, cells were lysed in IP-lysis buffer (50 mM Tris [pH 7.4], 150 mM NaCl, 0.5% Triton X-100) containing 1× protease inhibitor and 1 mM PMSF. The indicated portion of cell lysates was incubated with protein G magnetic beads prebound with rat anti-HA or mouse anti-FLAG antibody at 4°C for 1 h with rotation. After washing with IP-lysis buffer was performed, proteins bound to the beads were solubilized in SDS loading buffer by boiling for 5 min and were examined by Western blot (WB) analysis using the indicated antibody. The image is representative of results from two independent experiments with similar outcomes.

GRA35 localized on the PVM, where it might directly interact with host cytosolic NLRP1. Because cell death occurs rapidly after parasite invasion (5), it is difficult to detect a putative interaction between GRA35 and NLRP1 in parasite-infected macrophages. To investigate a direct interaction between Lewis rat NLRP1 and *Toxoplasma* GRA35, coimmunoprecipitation (co-IP) was performed in HEK293T cells transiently expressing FLAG-NLRP1 and GRA35-HA. The lysis of cotransfected cells was subjected to immunoprecipitation by using HA antibody and FLAG antibody. However, GRA35-HA was not detected in the FLAG-immunoprecipitated fraction, and FLAG-NLRP1 was not detected in the HA-immunoprecipitated fraction (Fig. 8). Thus, Lewis rat NLRP1 does not directly interact with *Toxoplasma* GRA35 in cotransfected HEK293T cells.

### *Toxoplasma* parasites deficient in GRA35, GRA42, or GRA43 do not establish a chronic infection in Lewis rats but have reduced fitness in F344 rats in which *Toxoplasma* does not activate the NLRP1 inflammasome.

**FIG 9:**
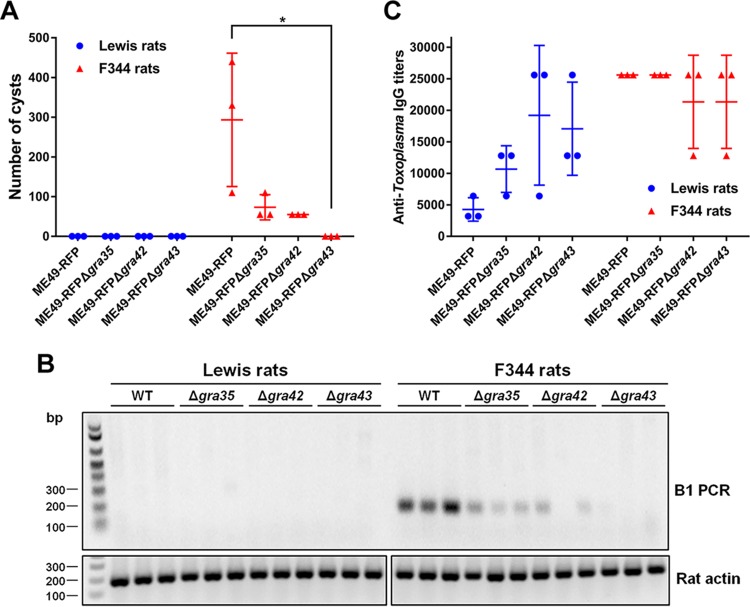
Parasites lacking GRA35, GRA42, and GRA43 do not establish a chronic infection in Lewis rats. (A) The number of brain cysts from each rat was determined by FITC-DBA staining at 60 days postinfection. Each plot represents the number of brain cysts of an individual rat (*n* = 3; *, *P < *0.05 [one-way ANOVA with Kruskal-Wallis test]). (B) The presence of *Toxoplasma* genomic DNA in the brain of infected rats was detected by diagnostic PCR targeting the multicopy *B1* gene. As an internal control, rat actin was used to check the quality of isolated DNA. The image is representative of results from two independent experiments. (C) The rat serum was obtained at 60 days postinfection. The anti-*Toxoplasma* IgG titers were quantified by ELISA. Titers were defined as the dilutions which gave an OD_405_ reading at least 2-fold higher than the mean background in uninfected rat serum. Results are presented as mean values ± SD obtained from individual infected rats (*n* = 3).

Since GRA35, GRA42, and GRA43 are required for parasite-induced macrophage pyroptosis *in vitro*, we hypothesized that *Toxoplasma* strains deficient in these genes would fail to induce macrophage cell death *in vivo*, allowing the parasite to replicate and eventually disseminate to the brain, leading to chronic infection. Removal of these genes does not lead to a general defect in parasite fitness in HFFs (32). We also found no significant difference in the levels of *in vitro* growth between WT parasites and Δ*gra35*, Δ*gra42*, or Δ*gra43* parasites in rat fibroblasts (Fig. S7A). Lewis rats were intraperitoneally (i.p.) infected with the type II ME49 strain expressing red fluorescent protein (RFP) or with the *GRA35*, *GRA42*, or *GRA43* knockout strains generated in this background. In addition, susceptible F344 rats, which encode an NLRP1 protein resistant to *Toxoplasma*-mediated inflammasome activation (2, 4), were used as a control. Compared to Lewis rat macrophages, F344 rat macrophages did not undergo rapid cell death after infection with WT, Δ*gra35*, Δ*gra42*, or Δ*gra43* parasites (Fig. S7B). During the course of infection, none of the rats lost weight or showed obvious clinical symptoms of toxoplasmosis (data not shown). After 2 months, the rats were sacrificed and the presence of cysts in the brains was determined. The brains of F344 rats infected with ME49-RFP parasites contained an average of 293 cysts, whereas, as expected, no detectable cysts were found in the brains of Lewis rats. F344 rats infected with Δ*gra35*, Δ*gra42*, or Δ*gra43* parasites contained reduced cyst numbers (73 cysts, 55 cysts, and 0 cysts on average per brain of rats infected with Δ*gra35*, Δ*gra42*, and Δ*gra43* parasites, respectively) (Fig. 9A). This suggests that GRA35, GRA42, and GRA43 determine *in vivo* fitness independently of their role in inflammasome activation. This was expected for GRA42 and GRA43, as Δ*gra42* and Δ*gra43* parasites have a defect in correct trafficking of GRAs to the PVM and because some PVM GRAs, such as GRA17, determine parasite fitness (31). The absence of parasites in the brain of Δ*gra43* parasite-infected F344 rats was confirmed by diagnostic PCR based on the *Toxoplasma B1* gene (Fig. 9B), which represents a repetitive sequence in its genome (33). Reduced cyst numbers in F344 rats could have been due to a defect of Δ*gra35*, Δ*gra42*, or Δ*gra43* parasites in cyst formation. However, Δ*gra35*, Δ*gra42*, and Δ*gra43* parasites formed normal *in vitro* cysts under alkaline stress induction conditions (Fig. S7C), suggesting that these GRAs play no role in cyst formation. Lewis rats infected with Δ*gra35*, Δ*gra42*, or Δ*gra43* parasites did not contain any brain cysts. Because GRA35, GRA42, and GRA43 determine *in vivo* fitness independently of their role in inflammasome activation, as shown by their defect in forming cysts in F344 rats, we cannot make conclusions on the contribution of NLRP1 inflammasome activation to Lewis rat sterile immunity to *Toxoplasma*.

10.1128/mBio.02388-18.7 FIG S7 GRA35, GRA42, or GRA43 mutants exhibit normal growth *in vitro*. (A) Confluent Brown Norway rat immortalized fibroblasts infected with type II WT parasites (ME49-RFP) or with the type II parasites in which *GRA35*, *GRA42*, or *GRA43* was knocked out (ME49-RFP Δ*gra35*, ME49-RFP Δ*gra42*, or ME49-RFP Δ*gra43*) for 7 days. The area of at least 40 plaques per experiment was measured. Data are displayed as average values with scatterplots for each independent experiment (*n *=* *3; error bars, ± SD; ns, not significant [one-way ANOVA with Kruskal-Wallis test]). (B) F344 rat BMDMs were infected with WT parasites or with parasites in which *GRA35*, *GRA42*, or *GRA43* was knocked out (Δ*gra35*, Δ*gra42*, or Δ*gra43*) or with knockout parasites complemented with WT alleles of *GRA35*, *GRA42*, or *GRA43* (Δ*gra35 *+* GRA35*, Δ*gra42 *+* GRA42*, or Δ*gra43 *+* GRA43*) (MOI = 1) for 24 h. Macrophage viability was measured via MTS assay. Data are displayed as a grouped column in combination with the Lewis rat cell viability data shown in Fig. 4C. Red indicates the average cell viability (+ SD) of Lewis rat BMDMs infected with the indicated parasites; blue indicates the average cell viability (+ SD) of F344 rat BMDMs infected with the indicated parasites (*n* = 3). (C) IFAs were performed on type II WT parasites (ME49-RFP) or the type II parasites in which *GRA35*, *GRA42*, or *GRA43* was knocked out (ME49-RFP Δ*gra35*, ME49-RFP Δ*gra42*, or ME49-RFP Δ*gra43*) as developing bradyzoite stages (for 3 days in alkaline [pH 8.2]). FITC-conjugated DBA was used to visualize the cyst wall. Images were taken at identical exposure times for each channel (scale bar = 10 μm). The image is representative of results from two independent experiments. Download FIG S7, TIF file, 1.1 MB. Copyright © 2019 Wang et al. 2019 Wang et al. This content is distributed under the terms of the Creative Commons Attribution 4.0 International license.

Although the Δ*gra35*, Δ*gra42*, or Δ*gra43* parasites seemed to be generally much less virulent than WT parasites in F344 rats, we hypothesized that their initial replication in macrophages in Lewis rats might still allow them to reach higher parasite numbers and dissemination levels than WT parasites. Previously, it was determined that higher parasite burdens in Lewis rats lead to higher anti-*Toxoplasma* antibody titers (2). We therefore compared the anti-*Toxoplasma* IgG titers in the sera obtained from all rats at 2 months postinfection (Fig. 9C). Lewis rats infected with ME49-RFP parasites had lower anti-*Toxoplasma* IgG titers (1/3,200 to 1/6,400) than F344 rats (titers ≥ 1/25,600). Lewis rats infected with Δ*gra35*, Δ*gra42*, or Δ*gra43* parasites had increased anti-*Toxoplasma* IgG titers (1/6,400 to 1/12,800, 1/6,400 to 1/25,600, or 1/12,800 to 1/25,600, respectively), whereas the titers were slightly decreased in F344 rats infected with Δ*gra42* or Δ*gra43* parasites (Fig. 9C). The increased titers of Lewis rats infected with Δ*gra35*, Δ*gra42*, or Δ*gra43*, compared to WT parasite-infected rats, might indicate that Δ*gra35*, Δ*gra42*, or Δ*gra43* parasites bypassed the NLRP1 inflammasome barrier in macrophages, allowing them to replicate and disseminate. We were unable to observe detectable IL-1β levels in the serum of parasite-infected Lewis rats and F344 rats regardless of parasite strain (data not shown). Taking the data together, even though deletion of *GRA35*, *GRA42*, and *GRA43* abrogates Lewis rat macrophage pyroptosis, thereby allowing Δ*gra35*, Δ*gra42*, and Δ*gra43* parasites to replicate inside Lewis rat macrophages *in vitro*, *Toxoplasma* parasites deficient in these genes still failed to develop cysts in the brain of Lewis rats. The most likely explanation is that these GRAs are also required for *in vivo* fitness independently of their role in Lewis rat inflammasome activation.

## DISCUSSION

We and others previously showed that *Toxoplasma* infection activates the NLRP1 inflammasome in Lewis rat macrophages, resulting in pyroptosis, secretion of mature IL-1β, and inhibition of parasite replication (4–6). This study showed that GRA35, GRA42, and GRA43 are parasite PV-localized proteins that mediate Lewis rat macrophage pyroptosis and secretion of mature IL-1β. The fact that Δ*asp5* parasites, but not Δ*myr1* parasites, no longer induced pyroptosis suggests that this cell death is mediated by PVM-localized GRAs. Several GRAs secreted onto the PVM have been identified as parasite effectors involved in host-parasite interactions, including modulation of host signaling pathways, evasion of host immune responses, and nutrition acquisition (23). GRA6 locates at the PVM, where it selectively activates the host transcription factor nuclear factor of activated T cells 4 (NFAT4) via interaction with host calcium modulating ligand (CAMLG) (34). GRA7 is a transmembrane protein that spans the PV and extends into the host cytosol, where it interacts with ROP complexes (35). GRA7 also binds directly to oligomers of the immunity-related GTPase Irga6, eventually leading to disassembly (35). GRA15 from type II *Toxoplasma*, another PVM-associated GRA, is involved in host NF-κB activation, which promotes the production of proinflammatory cytokines (36). Two additional dense granule proteins, GRA17 and GRA23, which are also located at the PV membrane, are responsible for small-molecule transport between the host cytosol and the vacuole lumen (31).

Although our results indicate that *Toxoplasma* GRAs localized to the PVM induced Lewis rat macrophage pyroptosis, the mechanism of inflammasome activation is still unclear. Cleavage of the NLRP1 N terminus is required for the activation of the inflammasome by anthrax LT (15). A recent study demonstrated that proteolysis can act as a common activator of diverse NLRP1 variants from mice and humans (37). The mechanism that emerges is that the N-terminal part, upon autocleavage of NLRP1 at the FIIND domain, inhibits the active C-terminal part of NLRP1, which contains the CARD domain. Therefore, degradation of this N-terminal part is needed for inflammasome activation (38, 39). GRA35, GRA42, and GRA43 have orthologues in *Hammondia*, *Neospora*, and *Besnoitia*. Neospora caninum is able to induce pyroptosis in Lewis rat macrophages (Fig. S8), suggesting that the mechanism of inflammasome activation is conserved in cyst-forming coccidia. GRA42 and GRA43 are mainly localized inside the PV, suggesting that they are the upstream mediators of inflammasome activation. As our results show that deletion of *GRA42* or *GRA43* affects the correct localization of other PVM GRAs, it is likely that GRA42 and GRA43 function as protein chaperones that help GRAs localize to the PVM, where GRA35 or another, unknown GRA then activates the NLRP1 inflammasome either directly or indirectly. The mutations of GRA35 in mutant 3 and mutant 4 are in the transmembrane domain, which results in GRA35 lacking its entire C terminus containing two coiled-coil domains. Coiled-coil domains function in many biological processes, including protein-DNA binding and protein-protein interaction (40). However, no direct interaction between Lewis rat NLRP1 and *Toxoplasma* GRA35 was found in cotransfected HEK293T cells. This suggests that GRA35 might also function as part of the GRA42/GRA43 complex upstream of the protein involved in NLRP1 inflammasome activation. Previously, we reported that parasite infection of murine macrophage cell lines or human fibroblasts stably expressing Lewis rat NLRP1 does not trigger cell death (5). This suggests that murine macrophages and human fibroblasts lack a factor needed for activation of the Lewis rat NLRP1 inflammasome by *Toxoplasma*. One hypothesis is that GRA35 or another, unknown PVM-localized GRA interacts with a rat-specific factor that subsequently mediates the activation of the NLRP1 inflammasome. This pattern has been demonstrated for GRA6, whose C terminus interacts with host cytosolic protein CAMLG, leading to NFAT4 activation (34). It is also possible that a *Toxoplasma* protein interacts with or modifies a Lewis rat-specific protein which is sensed by NLRP1, similarly to NLRC4 recognition of a NAIP5/NAIP6/flagellin complex (41, 42), or possibly inhibits the negatively regulation of NLRP1 by this rat factor. A further complication is that some inflammasomes do not directly interact with a pathogen-associated molecular pattern (PAMP) but rather sense changes to the cellular milieu induced by infection. For example, NLRP3 senses diverse cellular signals, such as K^+^ efflux, Ca^2+^ signaling, reactive oxygen species (ROS), mitochondrial dysfunction, and lysosomal rupture, which are the triggers for NLRP3 inflammasome activation (43). It is therefore possible that NLRP1 does not directly interact with a *Toxoplasma* effector but rather detects changes in the cell induced by *Toxoplasma* infection. For instance, cytosolic ATP depletion is sensed by NLRP1b, leading to inflammasome activation (44, 45). Another hypothesis is that GRA35 may also function as part of a putative GRA42/GRA43 complex that affects the PVM platform that supports or modifies other parasite effectors that somehow activate the NLRP1 inflammasome. This model has been described for the ROP5/ROP18/ROP17/GRA7 complex, which locates at the PVM and prevents PVM rupture by preventing the accumulation of immunity-related GTPases (IRGs) (35, 46).

10.1128/mBio.02388-18.8 FIG S8 Neospora caninum is able to induce cell death in Lewis rat macrophages. Lewis BMDMs primed with LPS (100 ng/ml, 2 h) or left untreated and infected with the indicated parasites (*Toxoplasma*, RH; *Neospora*, NC-1) at MOI =1 for 24 h. (A) Cell viability was measured using an MTS assay. (B) IL-1β secretion was measured using ELISA on cell supernatants. Data shown represent averages of results from two experiments, Error bars, + SD. Download FIG S8, TIF file, 0.4 MB. Copyright © 2019 Wang et al. 2019 Wang et al. This content is distributed under the terms of the Creative Commons Attribution 4.0 International license.

Although ASP5 influences GRA35 localization, there is no TEXEL motif present in GRA35 or in GRA42 and GRA43, suggesting that these three proteins are not direct substrates of ASP5. It is likely that another protein with a TEXEL motif mediates GRA35 localization to the PVM or functions as a regulator of GRA42 and GRA43 function. Identification of this protein could help us gain a better understanding of the mechanism of NLRP1 inflammasome activation.

Because GRA42 and GRA43 are important for correct localization of other GRAs at the PVM (Fig. 7B and C), some of which determine parasite fitness (31), it was expected that parasites lacking GRA42 or GRA43 would be less virulent *in vivo*. This was what we observed, as they had a defect in tissue cyst formation in susceptible F344 rats. However, this also made it difficult to establish the role of GRA42/GRA43-induced macrophage pyroptosis in Lewis rat sterile immunity. Unexpectedly, parasites lacking GRA35 were also unable to establish a chronic infection in Lewis rats. Because parasites lacking GRA35 also had a defect in tissue cyst formation in susceptible F344 rats, which possess a *Toxoplasma*-resistant variant of *Nlrp1*, and because F344 rat macrophages do not undergo pyroptosis *in vitro*, GRA35, like GRA42 and GRA43, must have an inflammasome-independent role in the pathogenesis of the parasite *in vivo*.

Despite the failure in tissue cyst formation, the higher anti-*Toxoplasma* IgG titers in the serum of Lewis rats infected with Δ*gra35*, Δ*gra42*, or Δ*gra43* parasites possibly indicate that pyroptosis was not induced during acute infection, allowing limited proliferation of tachyzoites, but that these parasites were eventually eliminated by other mechanisms. However, no parasites were detected in the peritoneal organs (spleen and liver) or the peritoneal cavity of Lewis rats and *Toxoplasma*-susceptible F344 rats by B1 sequence PCR and *in vivo* imaging at 2 days postinfection (data not shown), suggesting that, in general, the rat is resistant to the initial stage of infection. It remains unclear what mechanisms mediate parasite resistance in rats in which *Toxoplasma* does not activate the NLRP1 inflammasome (e.g., F344 rats).

Overall, the results presented here show that three dense granule proteins of Toxoplasma gondii are necessary for Lewis rat macrophage pyroptosis, which we have previously shown is induced by NLRP1 inflammasome activation (5). How these proteins function to activate the NLRP1 inflammasome is not yet known, but the data suggest a model where GRA42 and GRA43 mediate localization of GRA35 or of another, unknown GRA to the PVM, where it indirectly or directly mediates the activation of the NLRP1 inflammasome. Future experiments will be needed to determine the precise mechanism by which PVM-localized GRAs mediate the activation of the NLRP1 inflammasome and by which GRA42 and GRA43 influence the localization of GRAs to the PVM.

## MATERIALS AND METHODS

### Ethics statement.

All animal experiments were performed in strict accordance with the recommendations in the Guide for the Care and Use of Laboratory Animals of the National Institutes of Health and the Animal Welfare Act, approved by the Institutional Animal Care and Use Committee at the University of California, Davis (UC Davis) (assurance number A-3433-01).

### Reagents and antibodies.

ENU and EMS were purchased from Sigma-Aldrich. CellTiter 96 AQueous One Solution cell proliferation assay was obtained from Promega. Dextran sulfate sodium salt was obtained from Santa Cruz Biotechnology. LPS Escherichia coli O55:B5 (catalog no. 437625) was purchased from Calbiochem/EMD Biosciences. Caspase-1 inhibitor VX765 was purchased from Selleck Chemicals. NLRP3 inflammasome inhibitor MCC950 was purchased from AdipoGen Life Sciences, Inc. Nigericin (sodium salt) was purchased from MilliporeSigma. Rabbit anti-IL-1β (Ab9787) was purchased from Abcam. Rat anti-HA (3F10) antibody was obtained from Roche. Mouse anti-FLAG M2 antibody (F1804) was purchased from Sigma-Aldrich. Secondary horseradish peroxidase (HRP)-conjugated antibodies were purchased from Jackson ImmunoResearch. Alexa Fluor 448 and 594 secondary antibodies were obtained from Invitrogen.

### Rats and parasites.

Lewis (LEW/Crl; LEW) rats and F344 (F344/DuCrl; CDF) rats (6 to 8 weeks of age) were purchased from Charles River Laboratories (Wilmington, MA). Lewis rat bone marrow-derived macrophages (BMDMs) were prepared as previously described (5). Type I (RH) Toxoplasma gondii tachyzoites expressing luciferase and green fluorescent protein (GFP) were used for mutagenesis. RH parasites without luciferase and lacking the *HXGPRT* gene (RH Δ*hxgprt* parasites) were used for generating knockouts. RH parasites without luciferase and lacking the *HXGPRT* gene and the *Ku80* gene (RH Δ*hxgprt* Δ*ku80* parasites) were used as WT controls for Δ*asp5* parasites. Type II (ME49) parasites engineered to express RFP were a gift from Michael Grigg. RH Δ*sub1* parasites were a kind gift from Vern Carruthers and were generated as previously described (47). RH Δ*asp5* and RH-*ASP5*-Ty parasites were kind gifts from Mohamed-Ali Hakimi and were generated as previously described (26). All parasite strains were routinely passaged *in vitro* in monolayers of HFFs. PCR was used to confirm that all strains and cells were *Mycoplasma* negative.

### Mutagenesis screen.

Intracellular RH parasites expressing GFP and luciferase were treated with ENU (40 μM), EMS (100 μM), or DMSO for 4 h. Parasites were washed three times with phosphate-buffered saline (PBS), lysed by the use of a syringe, and allowed to infect fresh HFFs. For selection, Lewis BMDMs were infected with parasite populations (MOI = 0.2 to 0.3) for 2 h. Noninvading parasites were removed by washing cells with PBS three times. The medium was replaced with medium containing 30 mg/ml dextran sulfate. At 24 h postinfection, extracellular parasites were removed by washing cells with PBS three times. Cells were scraped into fresh media and overlaid onto fresh HFFs. After nine rounds of selection, parasites were cloned via serial dilution. Parasite DNA was isolated using a Qiagen DNeasy blood & tissue kit according to the manufacturer’s protocol. Illumina sequencing was performed on an Illumina HiSeq 2000 or MiSeq system. Reads were aligned using type I GT1 (v9.0) as the reference genome.

### Plasmid construction for ectopic expression and generation of complementation strains.

The plasmids were generated by cloning the gene with its putative promoter (∼2,000 bp upstream of the start codon) with a C-terminal hemagglutinin (HA) tag sequence into pENTR using TOPO cloning (Invitrogen) and then into pTKOatt using LR recombination (Invitrogen) (36). The primer sequences are available in Table S1.

10.1128/mBio.02388-18.9 TABLE S1 Sequences of primers used in this study. The HA tag sequence is bolded. Restriction enzyme sites are underlined. Download Table S1, XLSX file, 0.01 MB. Copyright © 2019 Wang et al. 2019 Wang et al. This content is distributed under the terms of the Creative Commons Attribution 4.0 International license.

### Generation of parasite strains.

Individual knockouts of candidate genes were performed using clustered regularly interspaced short palindromic repeat (CRISPR)-Cas9. Sequences targeting candidate genes were cloned into the pSS013-Cas9 vector (48). The sequences are available in Table S1. To generate the *MYR1* knockout strain and the knockout strains for the candidate hits from sequenced mutant clones, plasmids containing single guide RNA (sgRNAs) were cotransfected with XhoI (New England Biolabs)-linearized pTKOatt, which contains the *HXGPRT* selection cassette (36), into RH Δ*hxgprt* parasites at a 10:1 ratio (sgRNAs/linearized pTKOatt plasmid). At 24 h posttransfection, populations were selected with mycophenolic acid (50 μg/ml) and xanthine (50 μg/ml) and cloned by limiting dilution (Fig. S2A). Knockout was assessed by PCR (Fig. S2C). For generating complemented strains, knockout strains (Δ*gra35*, Δ*gra42*, or Δ*gra43* parasites) or mutant strains (mutant 1, mutant 2, or mutant 3) were cotransfected with the linearized complemented plasmid and a plasmid containing the dihydrofolate reductase (DHFR) resistance cassette at a ratio of 20:1. At 24 h posttransfection, populations were selected with pyrimethamine (1 μM) and cloned by limiting dilution. The presence of the tagged gene was determined by immunofluorescence assay (IFA) and Western blotting. To generate the double and triple knockout strains, Δ*gra35* parasites were cotransfected with separate plasmids containing sgRNAs against GRA42 or GRA43 together with NotI (New England Biolabs)-linearized pLoxp-DHFR-mCherry (49), which also contains a pyrimethamine resistance cassette, at a ratio of 5:1 (Fig. S2B). After two rounds of pyrimethamine selection and limiting dilution cloning, the double and triple knockout parasites were assessed by PCR and confirmed by sequencing in both loci. The GRA42 and GRA43 double knockout strain was generated from Δ*gra42* parasites by using a similar strategy. To generate each T. gondii *GT1_225160* (*TGGT1_225160*), *GRA36*, or *TGGT1_257970* knockout strain, plasmids containing sgRNAs were cotransfected with NotI (New England Biolabs)-linearized pLoxp-DHFR-mCherry at a ratio of 5:1 (Fig. S5A). After two rounds of pyrimethamine selection and limiting dilution cloning, the knockout parasites were assessed by PCR (Fig. S5B) and confirmed by sequencing.

### Cell viability analysis, counts of parasites per vacuole, and IL-1β quantitation.

Lewis rat BMDMs were incubated with or without 50 μM VX765 or 10 μM MCC950 for 2 h followed by parasite infection. F344 rat BMDMs were infected with parasites for 24 h. Cell viability was measured by 3-(4,5-dimethylthiazol-2-yl)-5-(3-carboxymethoxyphenyl)-2-(4-sulfophenyl)-2H-tetrazolium (MTS) assay as previously described (5). Counts of parasites per vacuole were performed as previously described (5). In LPS-primed BMDMs, the culture supernatants were collected for IL-1β quantitation by enzyme-linked immunosorbent assay (ELISA) as previously described (5). IL-1β in infected cell culture supernatants was also concentrated using Amicon filters (3-kDa molecular weight cutoff) (Millipore) and detected by Western blotting.

### Coimmunoprecipitation.

Plasmids expressing a C-terminal HA-tagged GRA35 without signal peptide (pcDNA3.1-GRA35-HA) and an N-terminal FLAG-tagged Lewis rat variant of NLRP1 (pCMV-FLAG-NLRP1) were mixed at a 1:1 ratio and transfected into HEK293T cells using X-tremeGENE 9 DNA transfection reagent (Roche) according to the manufacturer’s instructions. As controls, cells were also transfected with GRA35-HA plus FLAG empty vector and pcDNA3.1 empty vector plus FLAG-NLRP1 under the same conditions. After 30 h of transfection, cells were lysed in IP-lysis buffer (50 mM Tris [pH 7.4], 150 mM NaCl, 0.5% Triton X-100) containing 1× protease inhibitor and 1 mM phenylmethylsulfonyl fluoride (PMSF). The cell lysates were incubated with protein G magnetic beads prebound with rat anti-HA or mouse anti-FLAG antibody at 4°C for 1 h with rotation. After washing with IP-lysis buffer was performed, proteins bound to the beads were solubilized in SDS loading buffer by boiling for 5 min and examined by Western blot analysis. GRA35-HA was detected by rat anti-HA antibody, and FLAG-NLRP1 was detected by mouse anti-FLAG antibody.

### Western blotting.

To detect activated IL-1β, concentrated culture supernatants were separated on 12% SDS-PAGE gels and transferred to a polyvinylidene difluoride (PVDF) membrane (Bio-Rad, USA). To detect HA-tagged-GRA35, GRA42, or GRA43 expression, cell lysates made from intracellular parasites and extracellular parasites were separated onto 12% SDS-PAGE gels and transferred to a PVDF membrane. To detect interactions between GRA35-HA and FLAG-NLRP1, the coimmunoprecipitated samples were separated onto 12% SDS-PAGE gels and transferred to a PVDF membrane. Western blot analysis was performed as previously described (36).

### Invasion assay.

Lewis rat BMDMs were incubated with or without 50 μM VX765 or 10 μM MCC950 for 2 h followed by parasite infection. After 30 min of infection, a red/green invasion assay was performed as previously described (50) for indirect analysis of immunofluorescence.

### Immunofluorescent assay.

Extracellular parasites released from syringe-lysed HFFs were loaded onto coverslips and fixed with 100% ice-cold methanol for 5 min. Colocalization studies were performed with anti-GRA7 or anti-ROP1 and anti-HA antibodies. Alexa Fluor 488 and 594 secondary antibodies were used as previously described (36). To determine the localization of GRAs inside host cells, HFFs were infected with the different parasite strains for 24 to 30 h, fixed with 3% formaldehyde for 20 min, and permeabilized with 0.2% Triton X-100, followed by staining with rat anti-HA antibodies (1/500 dilution) or mouse monoclonal antibodies against *Toxoplasma* surface antigen (SAG1). Alexa Fluor 488 and 594 secondary antibodies were used as previously described (36).

### *In vitro* cyst induction.

Parasites were propagated in HFFs on coverslips under bradyzoite-inducing conditions (RPMI 1640 medium supplemented with 50 mM HEPES and 1% fetal bovine serum [pH 8.2], ambient CO_2_) for 3 days. Cells were then fixed with 100% ice-cold methanol and permeabilized with 0.2% Triton X-100, and the cysts were stained by the use of fluorescein isothiocyanate-Dolichos biflorus agglutinin (FITC-DBA) (Vector Laboratories).

### *In vivo* infection, cyst counting, diagnostic PCR, and serological detection.

*Toxoplasma* tachyzoites were harvested from cell culture and released by passage through a 27-gauge needle, followed by a 30-gauge needle. Three Lewis rats and three F344 rats (8 weeks of age) were infected intraperitoneally (i.p.) with 2 × 10^6^ parasites of each strain, and the parasite viability of the inoculums was determined in a plaque assay after infection. At 60 days postinfection, the rats were sacrificed and the brains were harvested. Following homogenization of brains by passage though a 21-gauge needle, cysts were stained by the use of FITC-DBA. To detect the presence of parasite in the brains of infected rats, genomic DNA of homogenized brains was isolated using Qiagen DNeasy blood & tissue kits (Qiagen). Diagnostic PCR targeting the B1 gene was performed using the primer sets listed in Table S1. To determine the anti-*Toxoplasma* IgG response of infected rats, serum was separated from blood obtained at 60 days postinfection and the anti-*Toxoplasma* IgG titer was determined using an enzyme-linked immunosorbent assay (ELISA). The plates were coated with 0.25 μg of whole-parasite lysate produced by several freeze-thaw cycles. After blocking with 2% bovine serum albumin (BSA)–PBS–0.05% Tween 20 was performed, serial dilutions of serum were added and the reaction mixtures were incubated at room temperature for at least 2 h, followed by incubation with 1/2,000-diluted HRP-conjugated goat anti-rat IgG at room temperature for 2 h. Finally, after washing with PBS–0.05% Tween 20 was performed, 100 μl of substrate solution {ABTS [2,2′-azinobis(3-ethylbenzthiazolinesulfonic acid)] solution; Sigma} was added to the wells, and after 30 min, the reaction was stopped by the addition of 50 μl of 0.3 M oxalic acid and the optical density at 405 nm (OD_405_) was measured. The titer corresponds to the dilution which gave an OD_405_ reading that was 2-fold higher than the average seen with uninfected rat serum.
