# Supplementary figures and images for: Three Toxoplasma gondii Dense Granule Proteins Are Required for Induction of Lewis Rat Macrophage Pyroptosis
Source: mBio. 2019 Jan 8;10(1):e02388-18. doi: 10.1128/mBio.02388-18 (PMC6325250; doi:10.1128/mBio.02388-18)

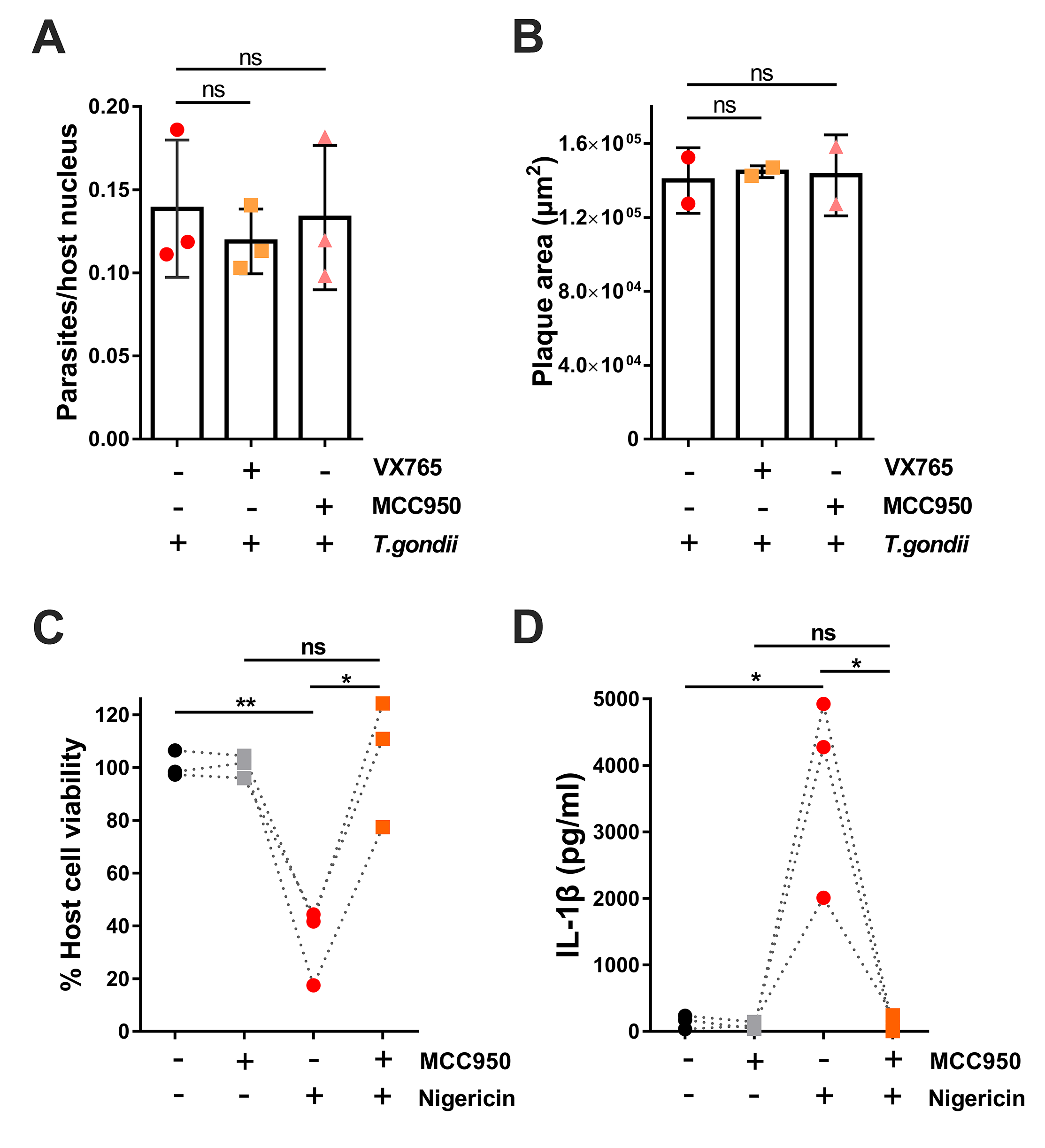

Supplement: FIG S1 [file mBio.02388-18-sf001.tif]

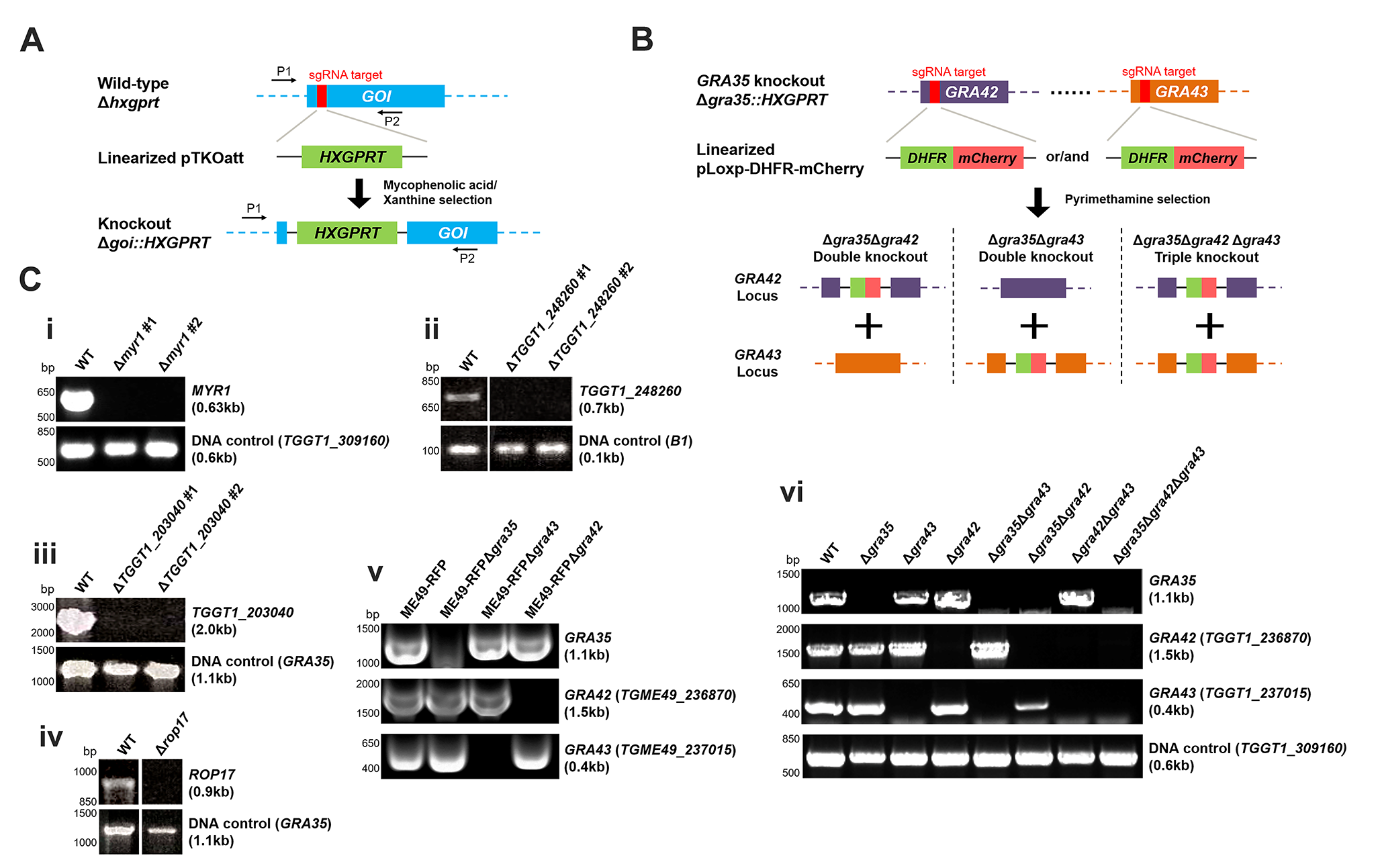

Supplement: FIG S2 [file mBio.02388-18-sf002.tif]

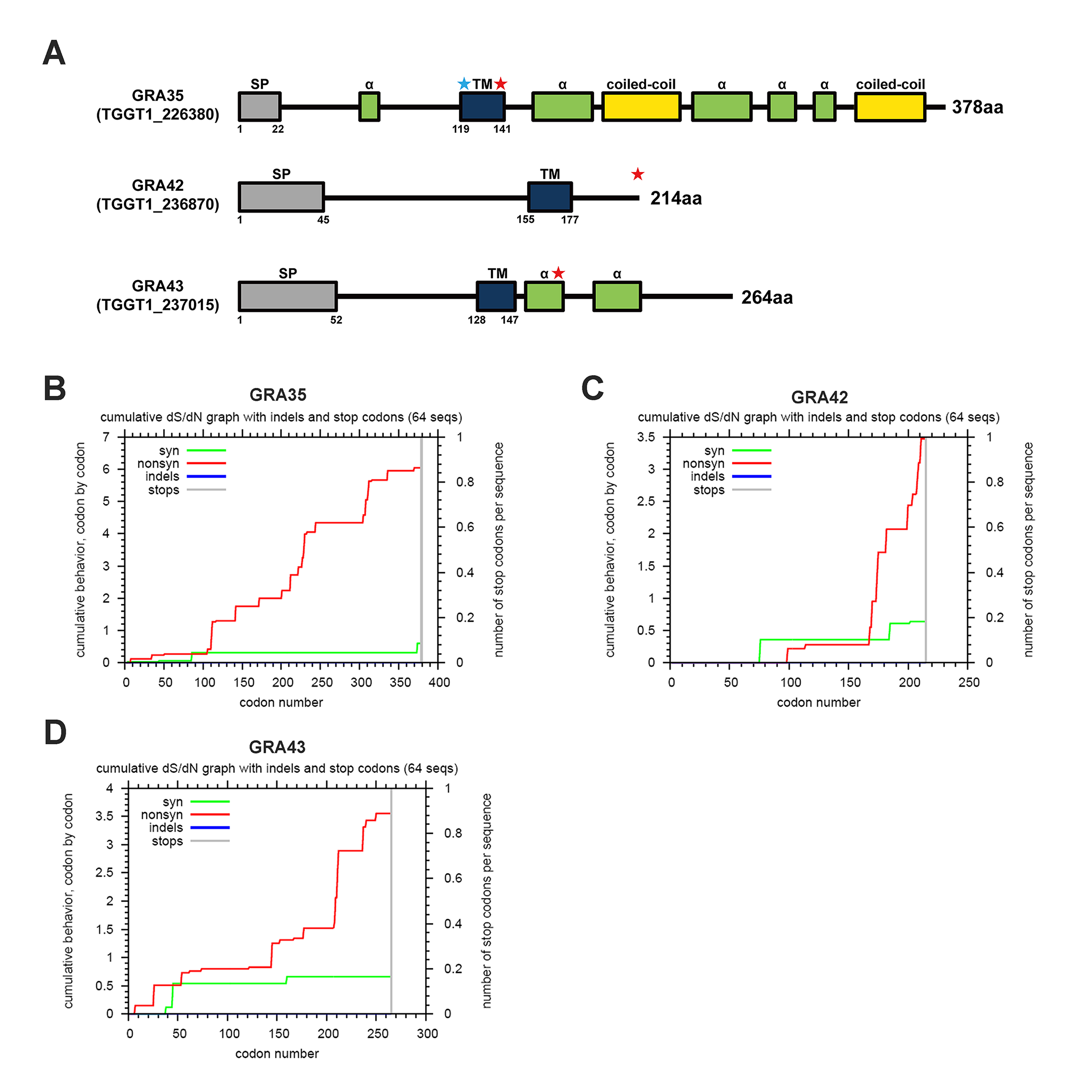

Supplement: FIG S3 [file mBio.02388-18-sf003.tif]

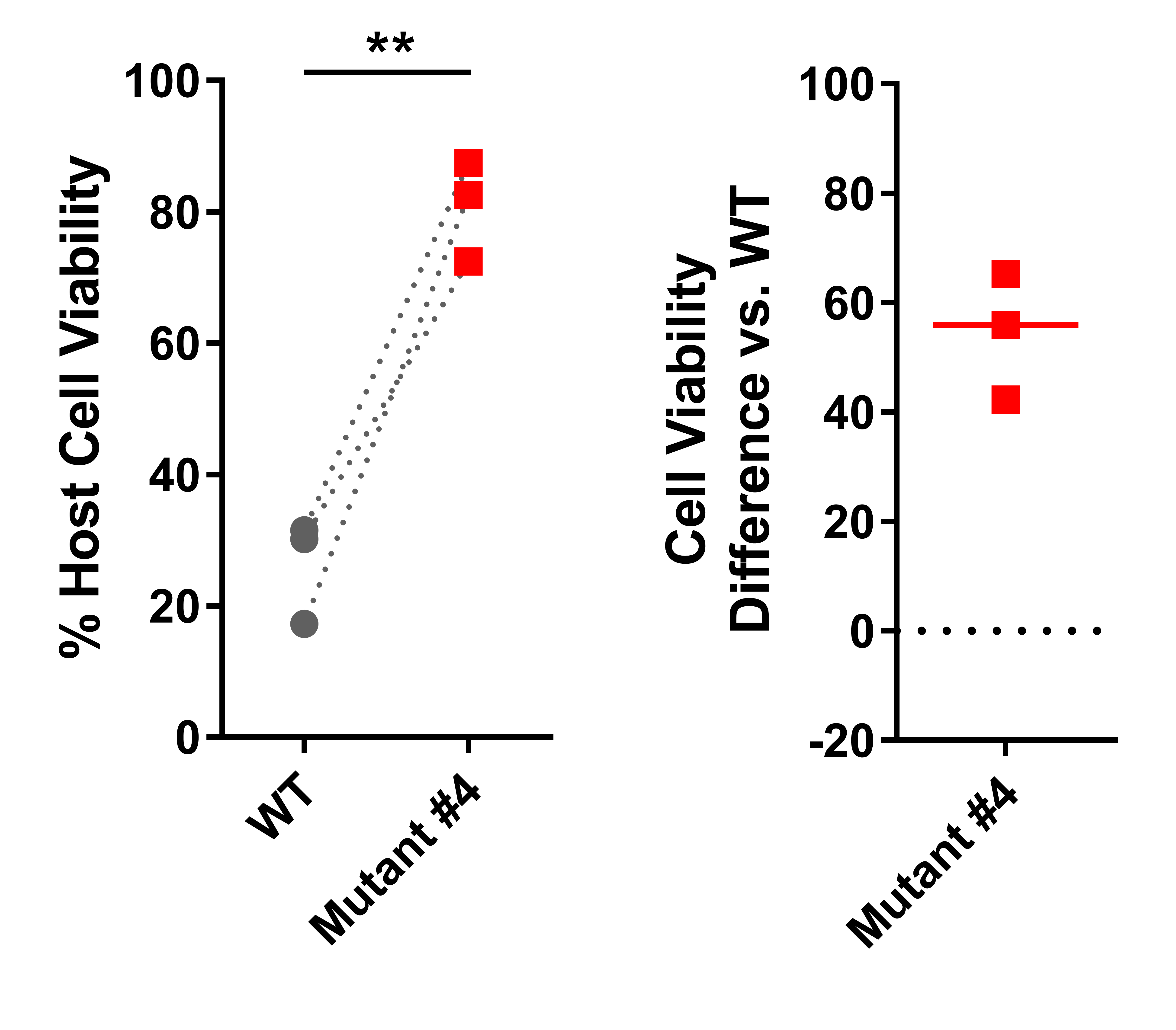

Supplement: FIG S4 [file mBio.02388-18-sf004.tif]

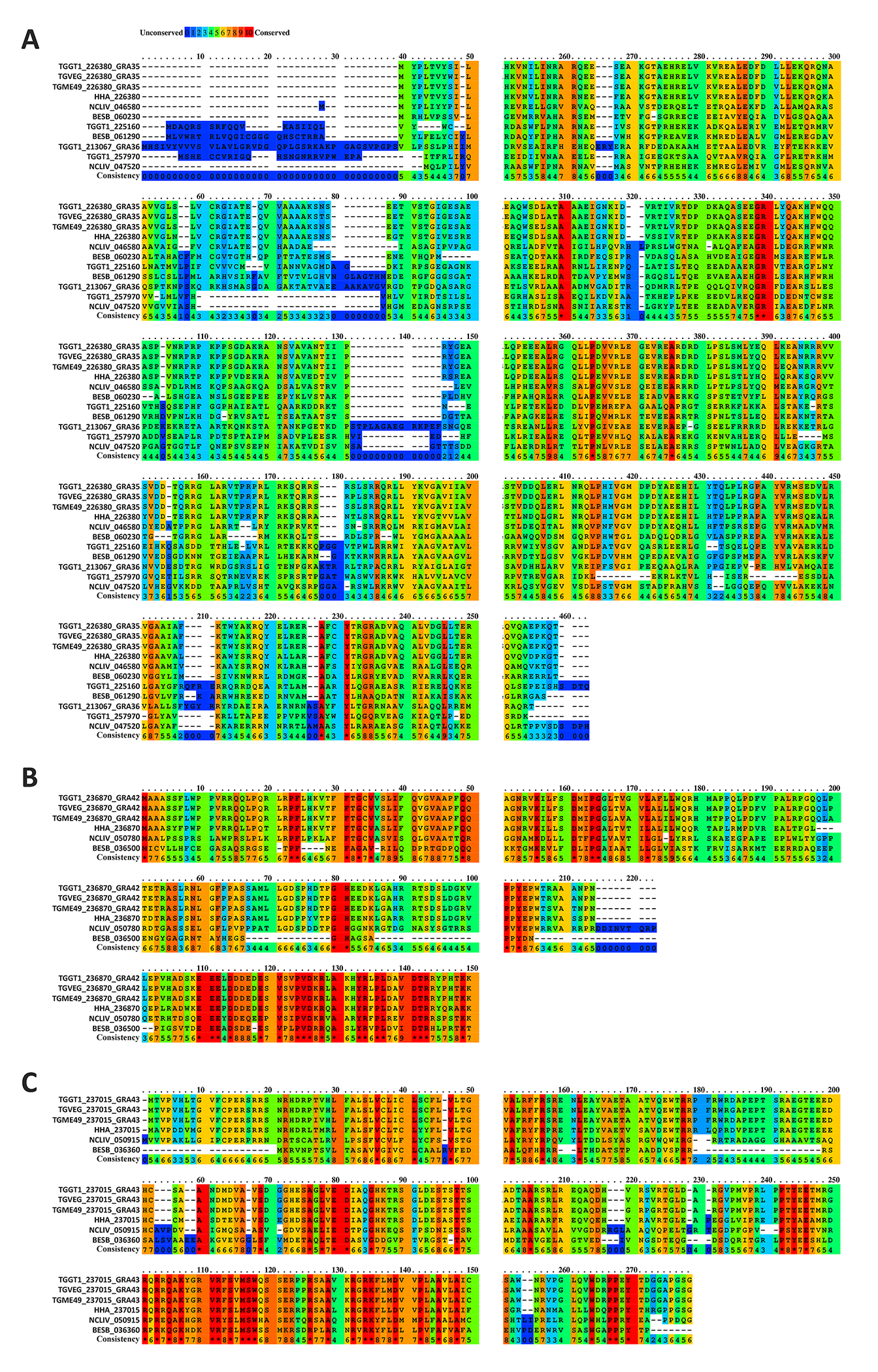

Supplement: FIG S5 [file mBio.02388-18-sf005.tif]

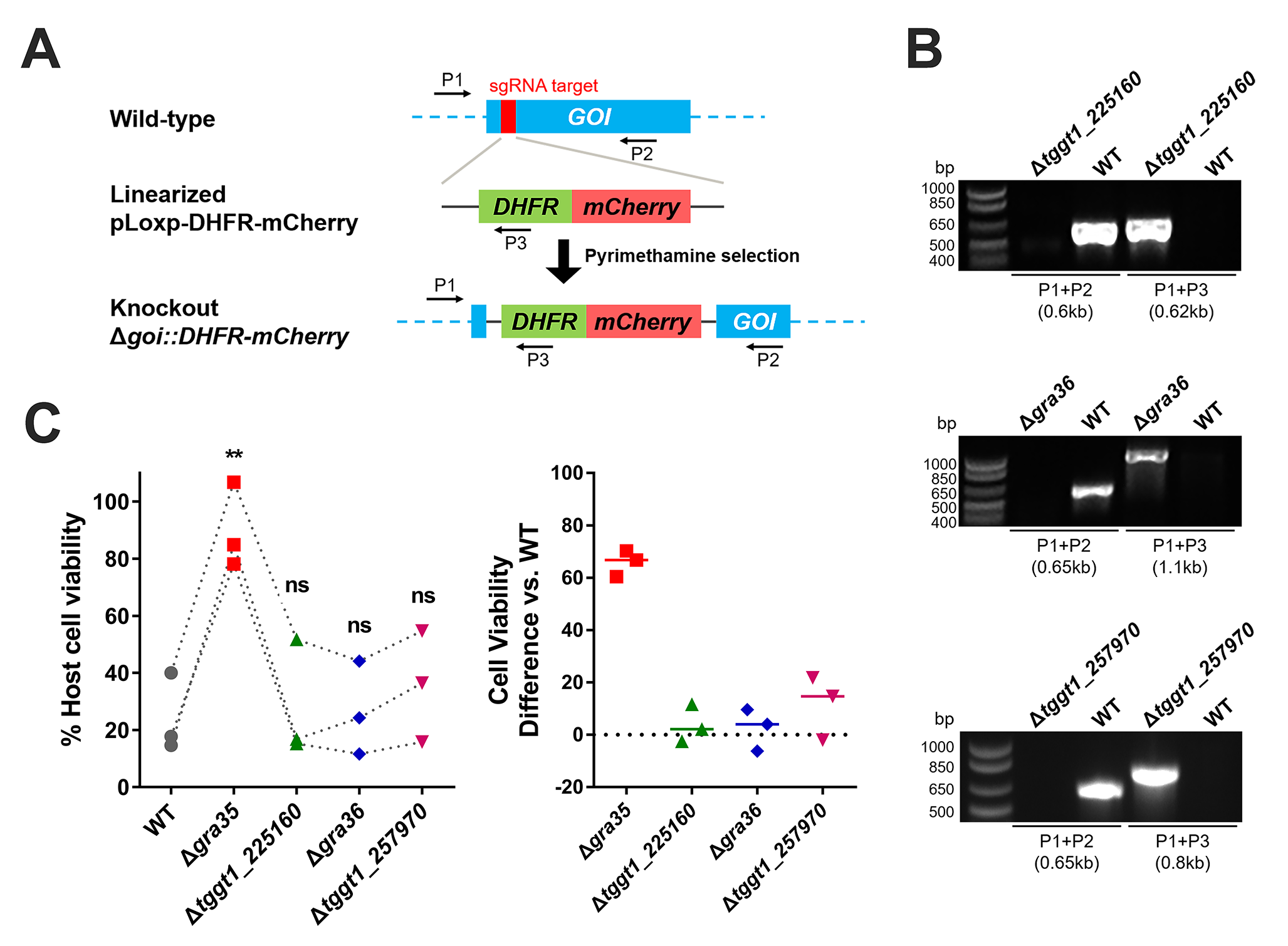

Supplement: FIG S6 [file mBio.02388-18-sf006.tif]

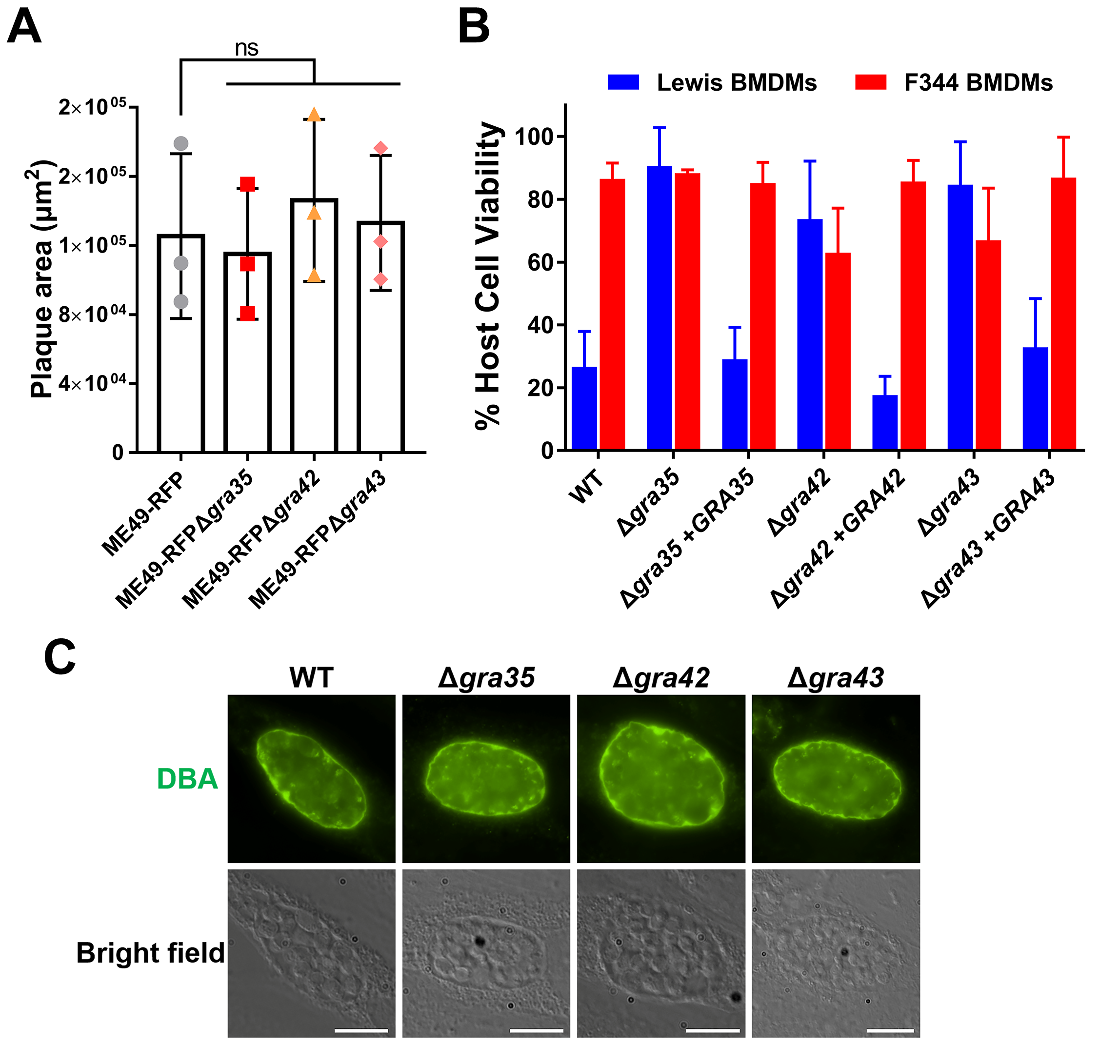

Supplement: FIG S7 [file mBio.02388-18-sf007.tif]

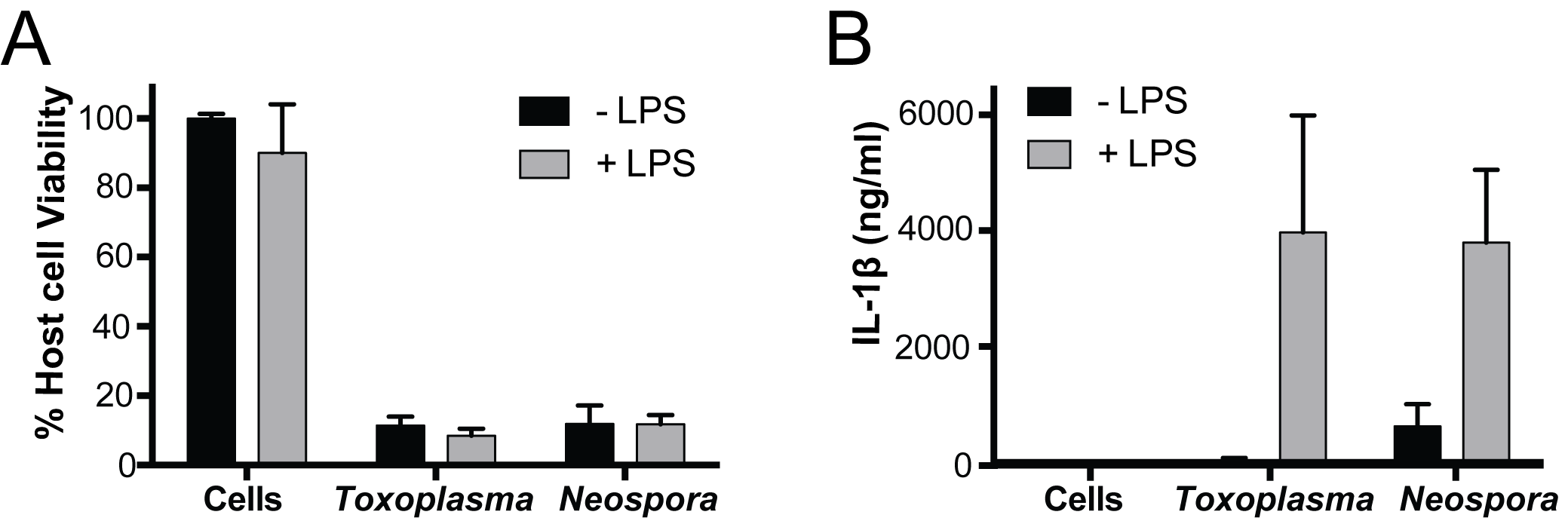

Supplement: FIG S8 [file mBio.02388-18-sf008.tif]
